# Design of a covalent protein-protein interaction inhibitor of SRPKs to suppress angiogenesis and invasion of cancer cells

**DOI:** 10.1038/s42004-024-01230-2

**Published:** 2024-06-27

**Authors:** Gongli Cai, Yishu Bao, Qingyun Li, Pang-Hung Hsu, Jiang Xia, Jacky Chi Ki Ngo

**Affiliations:** 1 School of Life Sciences, The Chinese University of Hong Kong, Shatin, N.T., Hong Kong SAR, China; 2 Department of Chemistry, The Chinese University of Hong Kong, Shatin, N.T., Hong Kong SAR, China; 3 Department of Bioscience and Biotechnology, National Taiwan Ocean University, Keelung, Taiwan; 4 Center of Excellence for the Oceans, National Taiwan Ocean University, Keelung, Taiwan; 5 Institute of Biochemistry and Molecular Biology, National Yang Ming Chiao Tung University, Taipei, Taiwan; 6 Center for Soybean Research of the State Key Laboratory of Agrobiotechnology, The Chinese University of Hong Kong, Shatin, N.T., Hong Kong SAR, China; 7 Center of Novel Biomaterials, The Chinese University of Hong Kong, Shatin, N.T., Hong Kong SAR, China; 8 Center for Protein Science and Crystallography, The Chinese University of Hong Kong, Shatin, N.T., Hong Kong SAR, China; 9 Present Address: College of Food Science and Engineering, Henan University of Technology, Zhengzhou, 450001 China

**Keywords:** Drug discovery and development, Pharmaceutics, Kinases, Chemical modification, Peptides

## Abstract

Serine–arginine (SR) proteins are splicing factors that play essential roles in both constitutive and alternative pre-mRNA splicing. Phosphorylation of their C-terminal RS domains by SR protein kinases (SRPKs) regulates their localization and diverse cellular activities. Dysregulation of phosphorylation has been implicated in many human diseases, including cancers. Here, we report the development of a covalent protein–protein interaction inhibitor, C-DBS, that targets a lysine residue within the SRPK-specific docking groove to block the interaction and phosphorylation of the prototypic SR protein SRSF1. C-DBS exhibits high specificity and conjugation efficiency both in vitro and *in cellulo*. This self-cell-penetrating inhibitor attenuates the phosphorylation of endogenous SR proteins and subsequently inhibits the angiogenesis, migration, and invasion of cancer cells. These findings provide a new foundation for the development of covalent SRPK inhibitors for combatting diseases such as cancer and viral infections and overcoming the resistance encountered by ATP-competitive inhibitors.

## Abstract (web summary)

The binding and phosphorylation of serine–arginine-rich (SR) proteins by SR protein kinases (SRPKs) is essential to regulate target gene expression, however, the efficient inhibition of this interaction and phosphorylation remains underexplored. Here, the authors develop a covalent inhibitor that targets the lysine residue within the SRPK-specific docking groove, to block interaction and phosphorylation of the prototypic SR protein SRSF1.

## Introduction

Serine–arginine-rich (SR) proteins are a family of non-small-nuclear ribonucleoprotein (non-snRNP) splicing factors that are essential for both constitutive and alternative splicing. They contain one or two RNA recognition motifs (RRMs) at their N-terminal regions and stretches of arginine–serine dipeptides at their C-terminal domains (RS domains). The reversible phosphorylation of these RS domains plays a pivotal role in regulating SR protein functions during spliceosome assembly, splice site selection, and mRNA metabolism^1–3^. Given their roles in multiple stages of gene expression, dysregulated expression or phosphorylation of SR proteins has been implicated in many human diseases^4,5^. SR protein kinases (SRPKs), one of the key kinase families that specifically phosphorylates SR proteins, are found in both the cytoplasm and nucleus and preferentially phosphorylate RS dipeptides in a regiospecific manner^6–8^. SRPKs phosphorylate multiple RS dipeptides within SR proteins to regulate their subcellular localization and functions during spliceosome assembly^9,10^. In particular, SRPK-mediated processive phosphorylation of the SR proteins SRSF1 and SRSF3 facilitates their nuclear import and dissociation from nuclear speckles, respectively^11,12^. In addition, the multisite phosphorylation of SRSF1 promotes the recruitment of U1-70K to the exonic splicing enhancer during formation of the spliceosome E complex^13^.

Given the pivotal roles of SRPKs in the regulation of SR splicing factors, their dysregulated expression can serve as predictive and prognostic indicators for various cancers^14–16^. High expression of SRPK1 or SRPK2 was observed in clinical samples of multiple carcinomas as well as nonepithelial cancers, such as glioblastomas and several forms of leukemia^15,17–19^. In some of these malignancies, increased kinase expression is correlated with higher tumor grade, a more advanced stage, higher metastatic potential, and shorter overall survival^15^. These findings highlight the potential of SRPKs as promising cellular targets for therapeutic interventions.

Several ATP-competitive SRPK-specific inhibitors, including the first reported SRPIN340, and SRPINX31, a potent inhibitor with high selectivity for SRPK1 over SRPK2, have been developed in the past decade^20,21^. In addition, we and our collaborators have reported the development of an irreversible inhibitor known as SRPKIN-1, which forms a covalent bond with a tyrosine residue at the ATP-binding pocket of SRPK1^22^. Although these compounds have been proven to be effective against SRPKs, certain concerns exist due to their ATP-competitive nature. For instance, ATP-competitive kinase inhibitors must compete with the high intracellular concentration of ATP. Potential drug polypharmacology will also arise because of the high structural similarity of the kinase ATP-binding pockets^23^. Moreover, many clinically approved ATP-competitive kinase inhibitors encounter drug-resistance-conferring mutations at ATP-binding pockets^24–27^. Thus, alternative strategies, such as targeting protein–protein interaction (PPI) surfaces, are preferred for the development of SRPK-specific inhibitors.

We previously demonstrated that the recognition and the processive multi-site phosphorylation of SRSF1 and SRSF3 by SRPKs are largely dependent on an SRPK-specific docking groove located at the C-terminal lobe of their kinase domains^11,12,28,29^. These findings indicate that the SRPK-specific docking groove may play a universal role in the binding and phosphorylation of SR proteins. On the basis of these findings, we previously developed a PPI inhibitor, DBS1, that targets this docking groove to inhibit both the binding and phosphorylation of SRSF1, and subsequently inhibits angiogenesis by switching the alternative splicing pattern of vascular endothelial growth factor (VEGF) from the proangiogenic isoform VEGF_165_a to the antiangiogenic isoform VEGF_165_b^30^. However, despite its specificity against SRPKs, DBS1 inhibited the SRPK1-mediated phosphorylation of SRSF1 with only a moderate IC_50_ value of 10.77 μM, indicating the need for further optimization of this inhibitor.

Covalent targeting is an effective strategy for the development of potent kinase inhibitors. Although cysteine is more commonly exploited, lysines are viable nucleophiles with the expansion of reactive warheads such as acrylamide, sulfone, phenyl ester, etc.^31–34^. In this study, we employed a structure-guided approach to modify DBS1 into a covalent inhibitor (named C-DBS) that targets the docking groove of SRPKs through proximity-enabled lysine-specific SuFEx reactions by introducing an aryl-sulfonyl fluoride group. C-DBS conjugated to a specific lysine within the docking groove and inhibited SRSF1 phosphorylation by SRPK1 with a significantly lower IC_50_ value compared with that for DBS1. In addition, C-DBS exhibited cell-penetrating ability and antiangiogenic and antimetastatic activities in cellular models. Our study highlights the potential of lysine residues in PPI interfaces as attractive targets for the development of covalent inhibitors.

## Results

### Development of DBS1 into a covalent inhibitor (C-DBS)

**Fig. 1:**
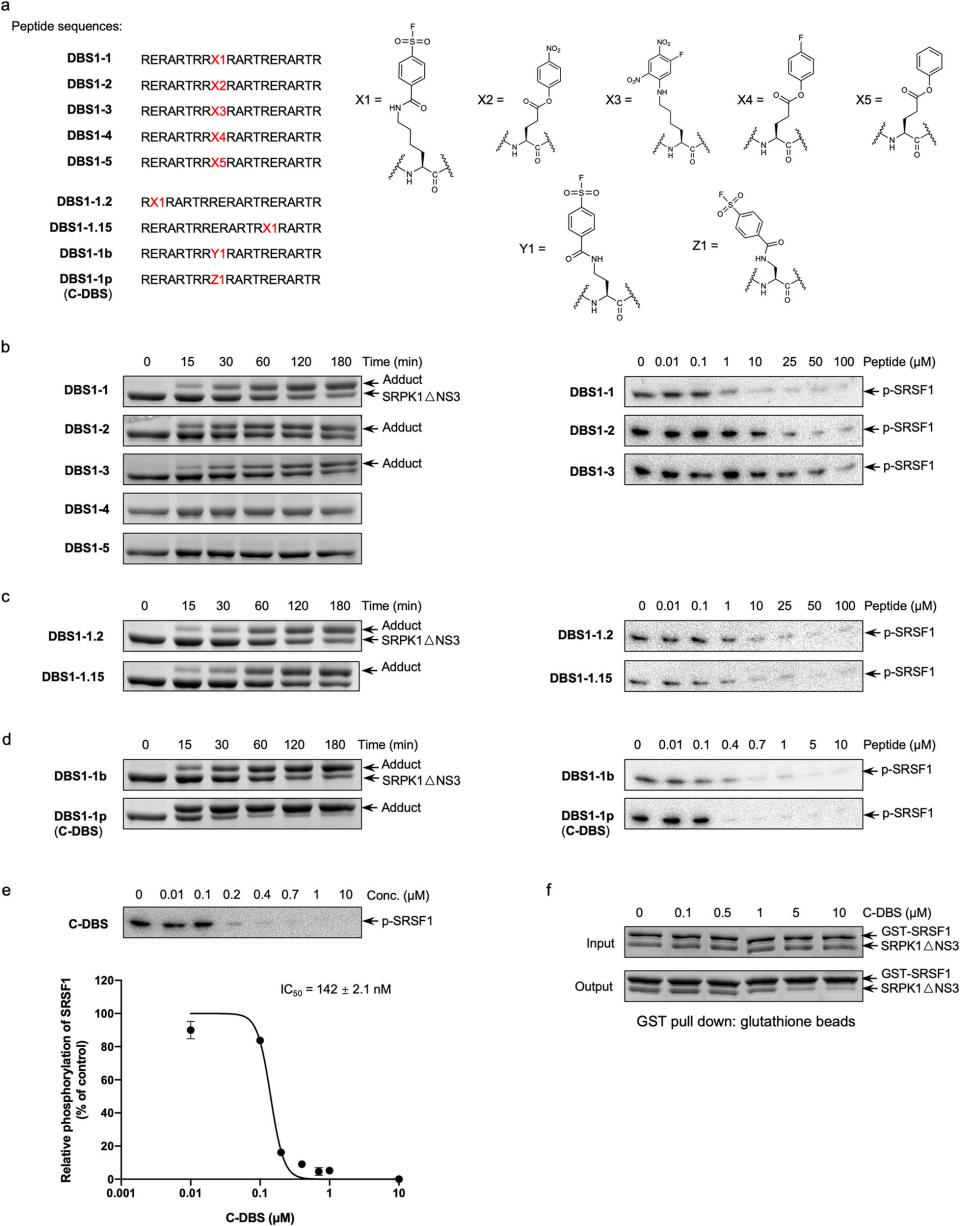
Rational design of an SRPK covalent inhibitor. **a** List of peptides designed with lysine-reactive modifications. The peptides comprise 20 amino acids with reactive chemical warheads installed. The chosen reactive groups were aryl-sulfonyl fluoride (X1), 4-nitrophenyl (X2), 1-fluoro-2,4-dinitrobenzene (FDNB) (X3), 4-fluorophenyl (X4), and phenyl (X5) groups. **b–d** Adduct formation assays (left panel) and in vitro kinase activity assays (right panel) of the modified peptides. The adduction assays were performed in a concentration of 1:4 (protein: peptide) for the indicated time periods. The samples were resolved by SDS-PAGE and visualized by Coomassie Blue. Kinase activity assays were performed using [^32^P]ATP in the presence of the modified peptides for 2 min. The reaction samples were resolved by SDS-PAGE and visualized by autoradiography. DBS1-1p (hereafter named as C-DBS) showed the best conjugation and inhibition of SRPK1. **e** C-DBS inhibited SRPK1-mediated SRSF1 phosphorylation with an IC_50_ value of 142 nM. Data represent means ± SEM from three independent experiments. **f** C-DBS disrupted the interaction between SRPK1 and SRSF1 dose-dependently. SRPK1 was preincubated with C-DBS at the indicated concentrations before the addition of GST-SRSF1. Samples were resolved by SDS-PAGE and visualized by Coomassie Blue.

Structural analysis of the SRPK1 docking groove revealed the presence of three solvent-exposed lysines (K602, K604, and K615) (Supplementary Fig. S1). These lysines are in proximity to arginines when an arginine-rich peptide binds to the docking groove. Therefore, we speculated that the apparent p*K*_a_s of the ε-amino groups of these lysines might decrease, rendering them more nucleophilic for lysine-reactive covalent conjugation. In addition, despite the absence of electron density for E2 in our SRPK1:7mer structure (PDB ID:7DD1), the side chain carboxyl group of E2 was predicted to be located in proximity to K602 and K604. Because our previous study demonstrated that the mid-region of DBS1 contributes most to its inhibitory activity^30^, we modified the E9 side chain of DBS1 (RERARTRRE^9^RARTRERARTR) with various reactive functional groups. Initially, we selected aryl-sulfonyl fluoride, 4-nitrophenol, fluorodinitrobenzene (FDNB), 4-fluorophenol, and phenyl groups as warheads and generated five derivative peptides (Fig. 1a). In brief, DBS1-1 and DBS1-3 were produced by replacing E9 with lysine and subsequently incorporating aryl-sulfonyl fluoride and FDNB, respectively. DBS1-2, DBS1-4 and DBS1-5 were generated by modifying E9 with nitrophenol, fluorophenol, and phenyl through esterification reactions between carboxyl and hydroxyl groups (Supplementary Fig. S2).

All derivative peptides were N-terminally acetylated and C-terminally amidated to ensure stability and prevent proteolysis. To examine the peptides’ conjugation ability with SRPK1, they were incubated with recombinant SRPK1ΔNS3, an active SRPK1 construct in which the nonconserved N terminus and spacer domain are truncated. Only three modified peptides—DBS1-1, DBS1-2, and DBS1-3—formed adducts with SRPK1ΔNS3 in a time-dependent manner, with DBS1-1 found to be the most efficient (Fig. 1b and Supplementary Data 1). Subsequently, we determined whether the three modified peptides could inhibit the phosphorylation of SRSF1 by SRPK1ΔNS3 (Fig. 1b and Supplementary Data 1). Our result showed that DBS1-1 exhibited the strongest inhibitory activity against SRPK1 with an IC_50_ value of 640 nM, which was a 17-fold improvement over that for DBS1 (IC_50_ = 11 μM) (Supplementary Fig. 3a ), demonstrating that the aryl-sulfonyl fluoride group is a suitable warhead for modifying DBS1 into a reactive peptide that targets SRPK1.

Despite the improvement achieved for DBS1-1, the reaction between excess derivative peptides and SRPK1ΔNS3 proteins did not reach completion even after prolonged incubation (Supplementary Fig. 3b). We speculated that positioning the warheads in different regions of the derivative peptides might improve the conjugation efficiency because the peptides might bind to alternative regions of the docking groove. We installed aryl-sulfonyl fluoride at positions 2 and 15 to generate DBS1-1.2 and DBS1-1.15, respectively (Fig. 1a). No difference was found between them in terms of the adduct formation efficiency or inhibitory activity (Fig. 1c, Supplementary Fig. S3b and Supplementary Data 1). Next, we investigated whether the sidechain length of lysine plays any role during the conjugation reaction. We replaced the lysine side chain at position 9 with lysine analogs diaminobutyric acid (Dab) and diaminopropionic acid (Dap) to generate DBS1-1b and DBS1-1p, respectively, and thereby shorten the distance between the peptide backbone and reactive site (Fig. 1a). These modifications enhanced the adduct formation efficiency. In particular, DBS1-1p, which contained the shortest sidechain, formed covalent adducts with nearly all SRPK1ΔNS3 after 60 min (Fig. 1d and Supplementary Fig. S3b). Kinase activity assays demonstrated that DBS1-1p inhibited the phosphorylation of SRSF1 by SRPK1 with an IC_50_ value of 142 nM, indicating a 75-fold improvement over the inhibition achieved with DBS1 (Fig. 1d, e, Supplementary Data 1, 2). This was equivalent to a *K*_i_ value of 104 nM obtained using the IC_50_-to-*K*_*i*_ converter developed by Lebeda and colleagues^35^. Subsequently, we investigated the inhibition mechanism of DBS1-1p using glutathione S-transferase (GST) pull-down assay, which revealed that DBS1-1p inhibited the interaction of SRPK1 with SRSF1 in a dose-dependent manner (Fig. 1f and Supplementary Data 1). We selected DBS1-1p as our covalent inhibitor candidate and hereafter refer to it as covalent docking blocker of SRPK (C-DBS) (Supplementary Fig. S3c). All C-DBS variants described in the following sections were purified through high-performance liquid chromatography and verified through mass spectroscopy (Supplementary Fig. S3d).

### C-DBS specifically conjugates to K604 at the docking groove

**Fig. 2:**
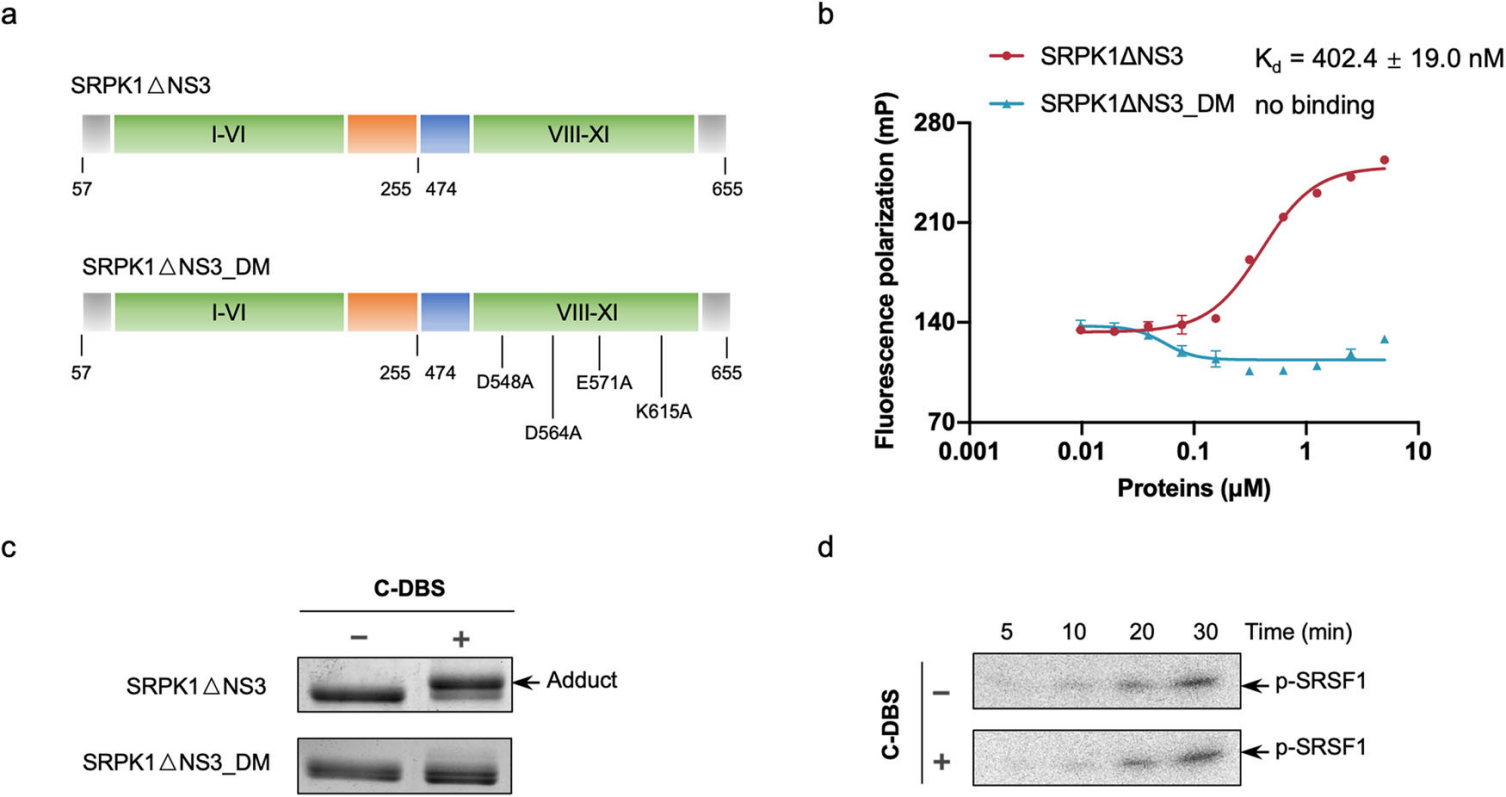
C-DBS targets the docking groove of SRPK1 and specifically conjugates to K604. **a** Schematic representation of the SRPK1ΔNS3 and SRPK1ΔNS3_DM constructs. Four residues (D548, D564, E571, and K615) that are essential to substrate binding and phosphorylation were mutated to alanines in SRPK1ΔNS3_DM. **b** The binding affinity between SRPK1ΔNS3 and C-DBS was measured using fluorescence polarization. A constant concentration (100 nM) of FAM-C-DBS titrated with varying concentrations of SRPK1ΔNS3, yielding a *K*_d_ value of 402 nM. Data represent means ± SEM from three independent experiments. **c** Adduct formation assays of SRPK1ΔNS3 and SRPK1ΔNS3_DM were performed in the absence or presence of C-DBS. SRPK1ΔNS3_DM failed to conjugate with C-DBS. **d** The presence of C-DBS did not alter the phosphorylation of SRSF1 by SRPK1ΔNS3_DM, which is incapable of processive phosphorylation of SRSF1.

Because DBS1 targets the docking groove of SRPK, we tested the specificity of C-DBS toward the same binding site. We performed fluorescence polarization (FP) assay to examine the direct noncovalent interaction between C-DBS and SRPK1ΔNS3, as well as the docking groove mutant SRPK1ΔNS3_DM that contains alanine substitutions at four docking groove residues—D548, D564, E571, and K615—that are essential for the binding and phosphorylation of the substrate (Fig. 2a)^11,12,36^. The apparent equilibrium dissociation constant *K*_d_ was 402 ± 19 nM (Fig. 2b and Supplementary Data 2), indicating that C-DBS bound SRPK1 with better affinity than did DBS1 (*K*_d_ = 3.2 ± 1.5 μM) prior to the covalent interaction. In contrast, SRPK1ΔNS3_DM failed to interact with C-DBS nor form adducts (Fig. 2b, c and Supplementary Data 1). Microscale thermophoresis assay (MST) also verified that the binding affinity of C-DBS was improved compared to DBS1, despite the obtained *K*_d_ value deviated from our FP result and IC_50_ value (Supplementary Fig. S4a). In vitro kinase assays further confirmed that the phosphorylation of SRSF1 by SRPK1ΔNS3_DM, which was weakened compared to that by SRPK1ΔNS3 (Supplementary Fig. S4b), was not affected by C-DBS because it failed to bind to the docking groove (Fig. 2d and Supplementary Data 1). These findings indicated that the inhibitor specifically targeted the docking groove to inhibit SRSF1 phosphorylation.

**Fig. 3:**
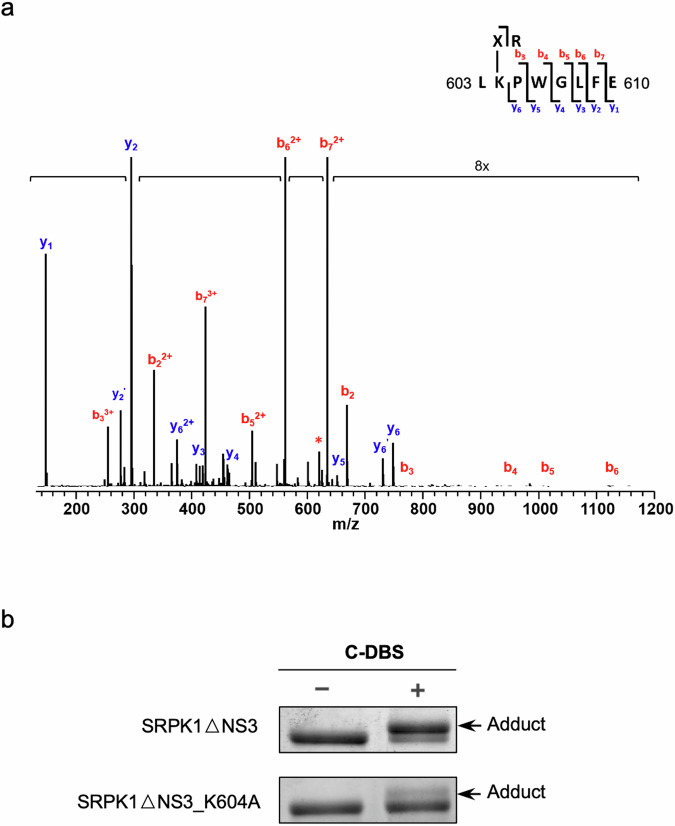
C-DBS specifically conjugates to K604. **a** MS2 analysis of C-DBS labeled SRPK1ΔNS3 identified K604 at the docking groove was the residue covalently conjugated with C-DBS. The adduct band in SDS-PAGE was excised and digested by trypsin and Glu-C overnight for mass analysis. **b** Adduct formation assay of SRPK1ΔNS3 and SRPK1ΔNS3_K604A with C-DBS. The amount of the adduct formed with SRPK1ΔNS3_K604A was significantly reduced compared to that of SRPK1ΔNS3.

Next, we attempted to identify lysine residues to which C-DBS was conjugated. Excised adduct bands of SRPK1ΔNS3 and C-DBS were in-gel digested by using trypsin/Glu-C. MALDI-TOF/TOF analysis was performed on the C-DBS-conjugated SRPK1 and on the SRPK1 alone. The experimental MS2 mass spectra revealed the presence of a peptide fragment LK(X-R)PWGLFE, where X represents Dap-aryl-SO_2_F in the SRPK1-C-DBS adduct sample but not in the SRPK1 alone sample. This identified peptide corresponded to residues 603 to 610 of SRPK1, and the labeled lysine was confirmed to be K604 (Fig. 3a), demonstrating that K604 was the conjugation site. Subsequently, we directly mutated K604 to Ala and repeated the adduction assay. The quantity of adducts formed with the SRPK1ΔNS3_K604A mutant was significantly lower compared with that in the reaction with SRPK1ΔNS3, thus confirming that K604 is the primary target site for C-DBS (Fig. 3b and Supplementary Data 1).

### C-DBS is a highly specific and selective pan-SRPK inhibitor

**Fig. 4:**
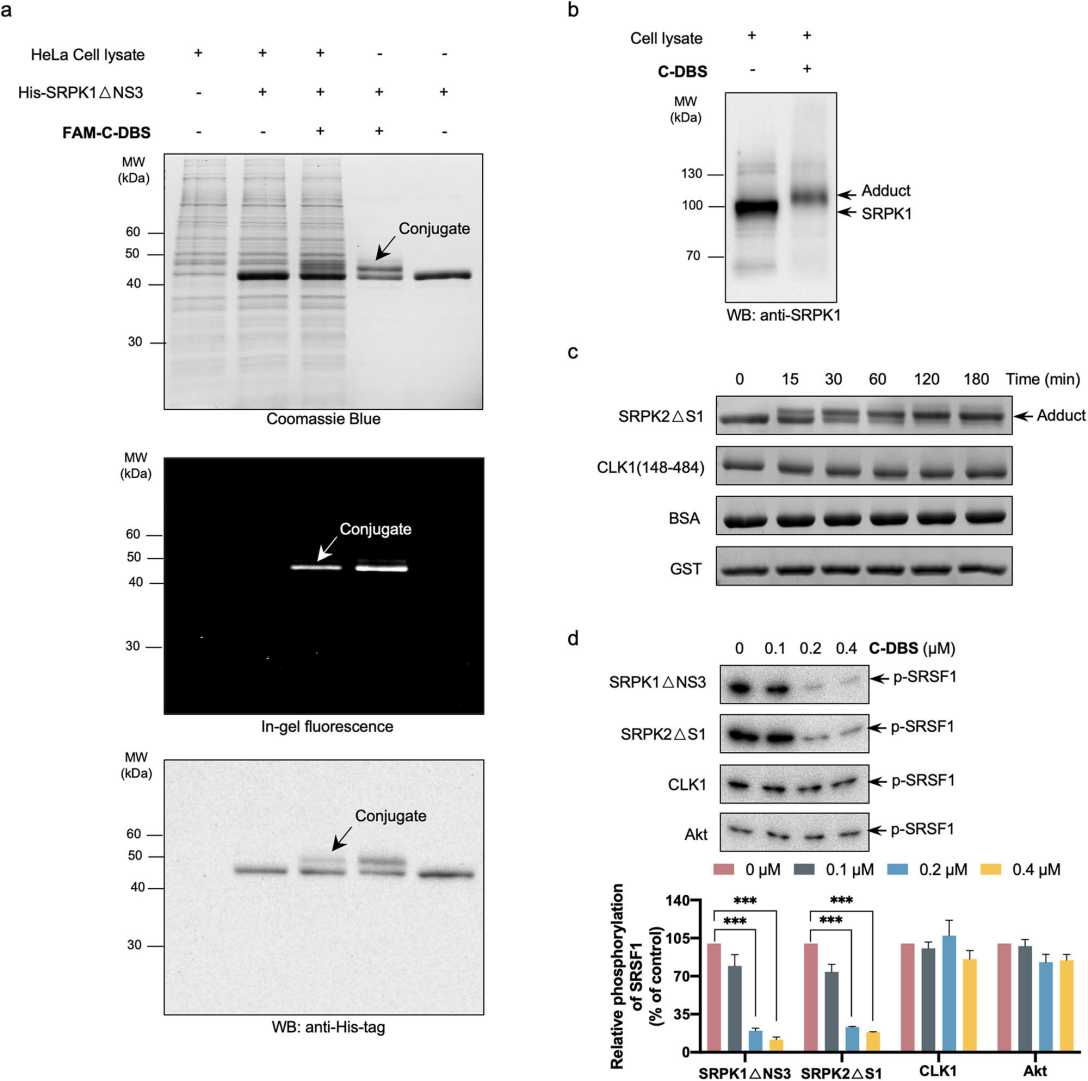
C-DBS is a selective pan-SRPK inhibitor. **a** FAM-C-DBS conjugated with recombinant His-tagged SRPK1ΔNS3 in the absence or presence of HeLa cell lysate. The samples were resolved by SDS-PAGE and the adduct bands were visualized using Coomassie Blue (top panel), in-gel fluorescence (middle panel), and Western blotting (bottom panel). **b** C-DBS conjugated with endogenous SRPK1. MDA-MB-231 cell lysate samples before and after incubation with C-DBS were resolved by SDS-PAGE. SRPK1 and the adducts were detected using the anti-SRPK1 antibody. **c** C-DBS conjugated with SRPK2ΔS1. C-DBS was incubated with SRPK2ΔS1, CLK1 kinase domain, BSA, and GST for the indicated time periods. Only SRPK2 formed adducts. **d** C-DBS selectively inhibited SRPKs. Kinase activity assays were performed toward SRPK1ΔNS3, SRPK2ΔS1, CLK1, and Akt in the presence of the indicated concentrations of C-DBS. C-DBS inhibited both SRPK1 and SRPK2 but not CLK1 or Akt. The p-SRSF1 levels were quantified using ImageJ. Data represents means ± SEM from three independent experiments. Statistical analysis was performed using one-way ANOVA. ***p < 0.001.

Since the docking groove is unique for SRPKs, we examined the specificity of C-DBS by performing the adduction assay in the presence of endogenous proteins. Recombinant His-tagged SRPK1ΔNS3 proteins were incubated with 6-carboxyfluorescein (FAM)-labeled C-DBS (FAM-C-DBS) in the absence or presence of HeLa cell lysate and were resolved through SDS-PAGE. New bands corresponding to the adducts were clearly visualized after Coomassie blue staining, regardless of the presence of the HeLa cell lysate (Fig. 4a and Supplementary Data 1). Fluorescence imaging and Western blotting of the samples with anti-His antibody further confirmed that the new bands represented SRPK1-C-DBS adducts (Fig. 4a and Supplementary Data 1). Then, we incubated C-DBS with MDA-MB-231 cell lysate alone to determine whether it interacts with endogenous SRPK1. Western blotting results revealed that the band corresponding to endogenous SRPK1 was shifted upward after the addition of C-DBS, suggesting that the inhibitor effectively formed adducts with the endogenous kinase (Fig. 4b and Supplementary Data 1).

Both SRPK1 and SRPK2 contain lysine residues in the same position at their docking grooves (K604 and K648 in SRPK1 and SRPK2, respectively) (Supplementary Fig. S5a). Therefore, like DBS1, C-DBS may be a pan-SRPK inhibitor that can inhibit SRPK2 through adduction. We examined the selectivity of C-DBS toward SRPK2, another SR protein-specific kinase CLK1, and two other unrelated proteins (bovine serum albumin [BSA] and GST). The results revealed that C-DBS effectively formed adducts with SRPK2 only, indicating that C-DBS selectively targets SRPKs (Fig. 4c and Supplementary Data 1). We investigated the inhibitory activity of C-DBS against kinases that phosphorylate SR proteins, including SRPK2, CLK1, and Akt, and determined that C-DBS selectively targets SRPKs (Fig. 4d and Supplementary Data 1, 2). The selectivity of C-DBS was further assessed by screening its inhibitory activity against 140 kinases. Although C-DBS was highly selective and did not significantly inhibit all the protein kinases tested, it also did not inhibit SRPK1 in the screen (Supplementary Fig. S5b). This was expected because free RS-rich peptides instead of the full SRSF1 substrate were used in this assay. Therefore, C-DBS, which targets the docking groove distal to the active site, could not inhibit phosphorylation of the free peptide.

### C-DBS is self-cell-permeable

**Fig. 5:**
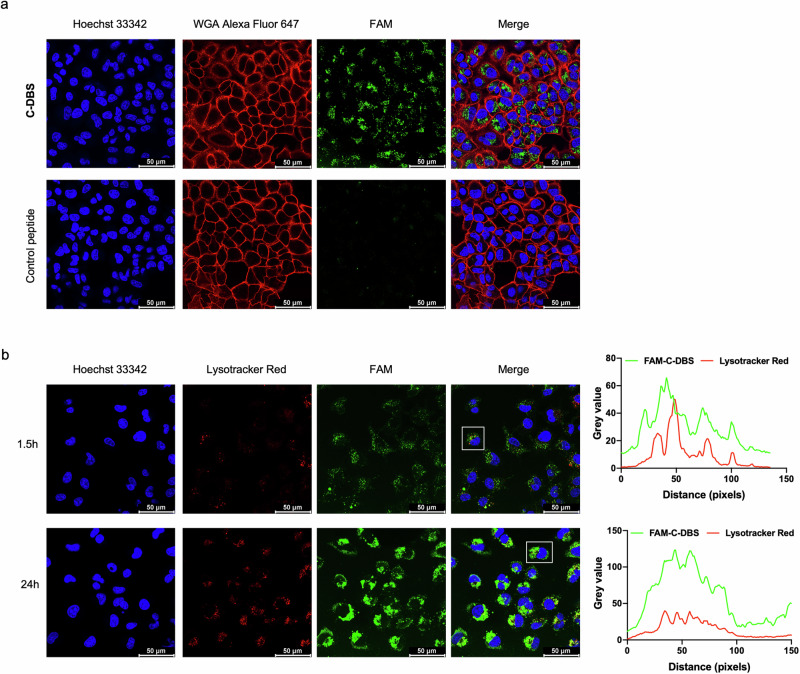
C-DBS is self-cell permeable. **a** Internalization of C-DBS and a control peptide in A549 cells. Both peptides were labeled with fluorescent FAM group (green). The cells were treated with 10 μM peptides for 24 h. Hoechst 33342 (blue) and Wheat Germ Agglutinin (WGA) Alexa Fluor 647 (red) were used to stain the nucleus and cell membrane, respectively. C-DBS, but not the control peptide, was readily up-taken by the cells. **b** Fluorescence analysis of FAM-C-DBS and Lysotracker. A549 cells were stained with Hoechst 33342 (blue) and Lysotracker Red (red) after C-DBS incubation for the indicated time. Increased diffused green signals were observed after prolonged incubation. The fluorescence intensity profiles of the region of interest (ROI, white box) were analyzed using ImageJ.

The arginine-rich sequence of C-DBS resembles that of cell-penetrating peptides (CPPs). To determine whether C-DBS exhibits cell-penetrating capability, we treated human non-small cell lung cancer (NSCLC) A549 cells with FAM-C-DBS. Confocal fluorescence microscopy revealed that C-DBS was readily internalized by the A549 cells. However, a FAM-labeled reactive control peptide consisting of an irrelevant amino acid sequence (LNGHEDAQ(Dap-aryl-SO_2_F)FPTRIVYLSKM) failed to enter the cells (Fig. 5a). The cellular uptake of C-DBS was further investigated by staining cells with LysoTracker Red after 1.5 or 24 h of incubation to determine whether the inhibitor colocalizes with endocytic vesicles. At 1.5 h, some C-DBS was observed to be trapped inside endosomal/lysosomal compartments, whereas the remaining C-DBS was noted in the cytoplasm. The signals of C-DBS were more diffused and more visible in the cytoplasm when incubation was performed for 24 h. Plots of the fluorescence intensities of C-DBS and LysoTracker Red revealed that the two did not overlap perfectly and that the mismatch was more obvious for the longer incubation time (Fig. 5b and Supplementary Data 2). These findings indicated that C-DBS was not trapped in endolysosomal compartments after internalization. Together, our data support the notion that C-DBS can be taken up by cells without any additional assistance.

### C-DBS inhibits SR protein phosphorylation, angiogenesis and cell invasion

**Fig. 6:**
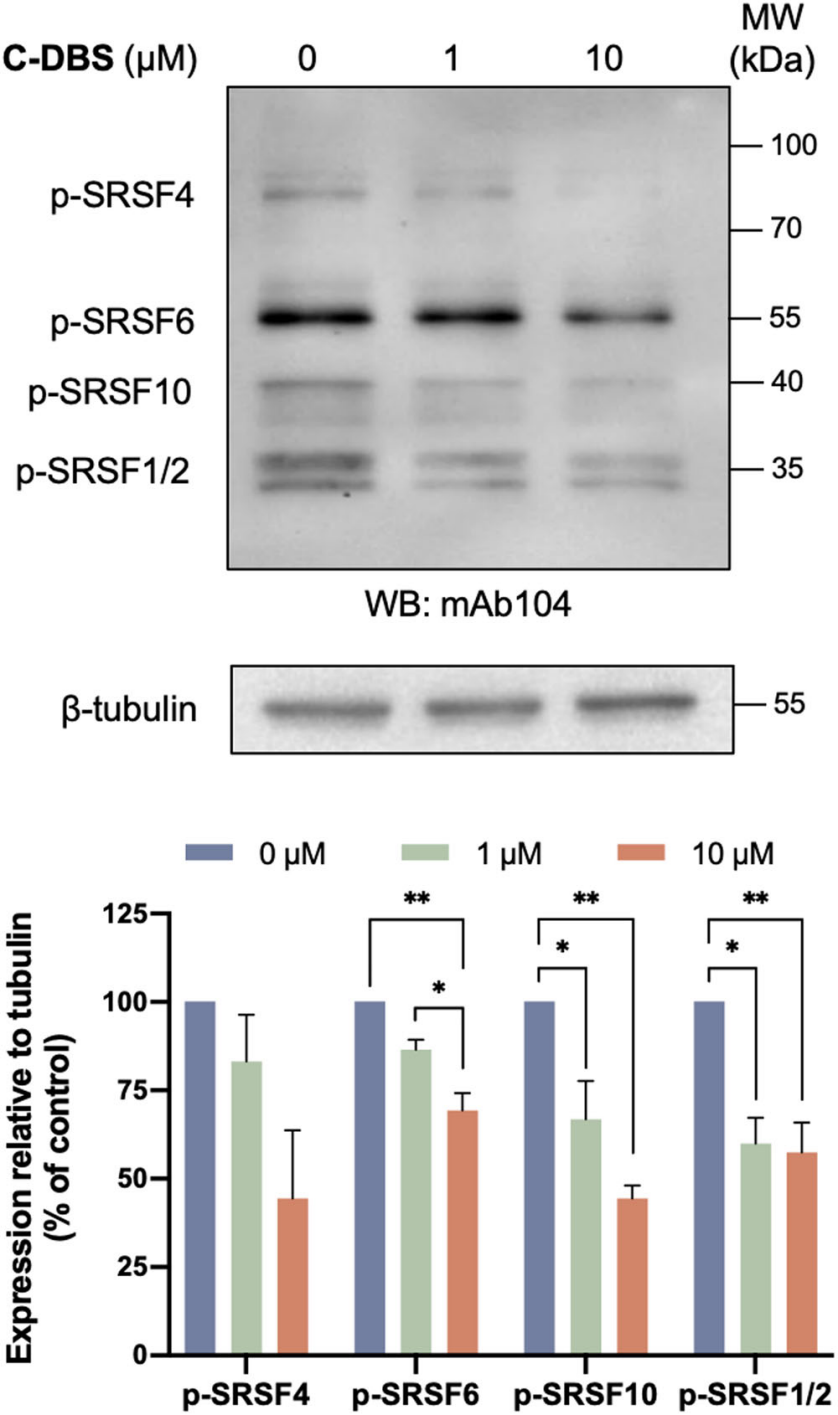
C-DBS inhibits SR protein phosphorylation. A549 cells were treated with the indicated concentrations of C-DBS and the total cell lysate was subjected to Western blotting, where the phosphorylated SR proteins were probed with mAb104. The p-SR levels versus β-tubulin were quantified using ImageJ. Data represents means ± SEM from three independent experiments. Statistical analysis was performed using one-way ANOVA. *p < 0.05; **p < 0.01.

Next, we evaluated the phosphorylation levels of SR proteins in A549 cells after treatment with different dosages of C-DBS. The cell lysates were analyzed using the phosphor-SR protein-specific mAb104 antibody^37^. The phosphorylation of several SR proteins—including SRSF1 or SRSF2, SRSF4, SRSF6, and SRSF10—was significantly attenuated by C-DBS dose-dependently (Fig. 6 and Supplementary Data 1, 2), demonstrating that C-DBS effectively inhibited the phosphorylation of endogenous SR proteins in cells.

**Fig. 7:**
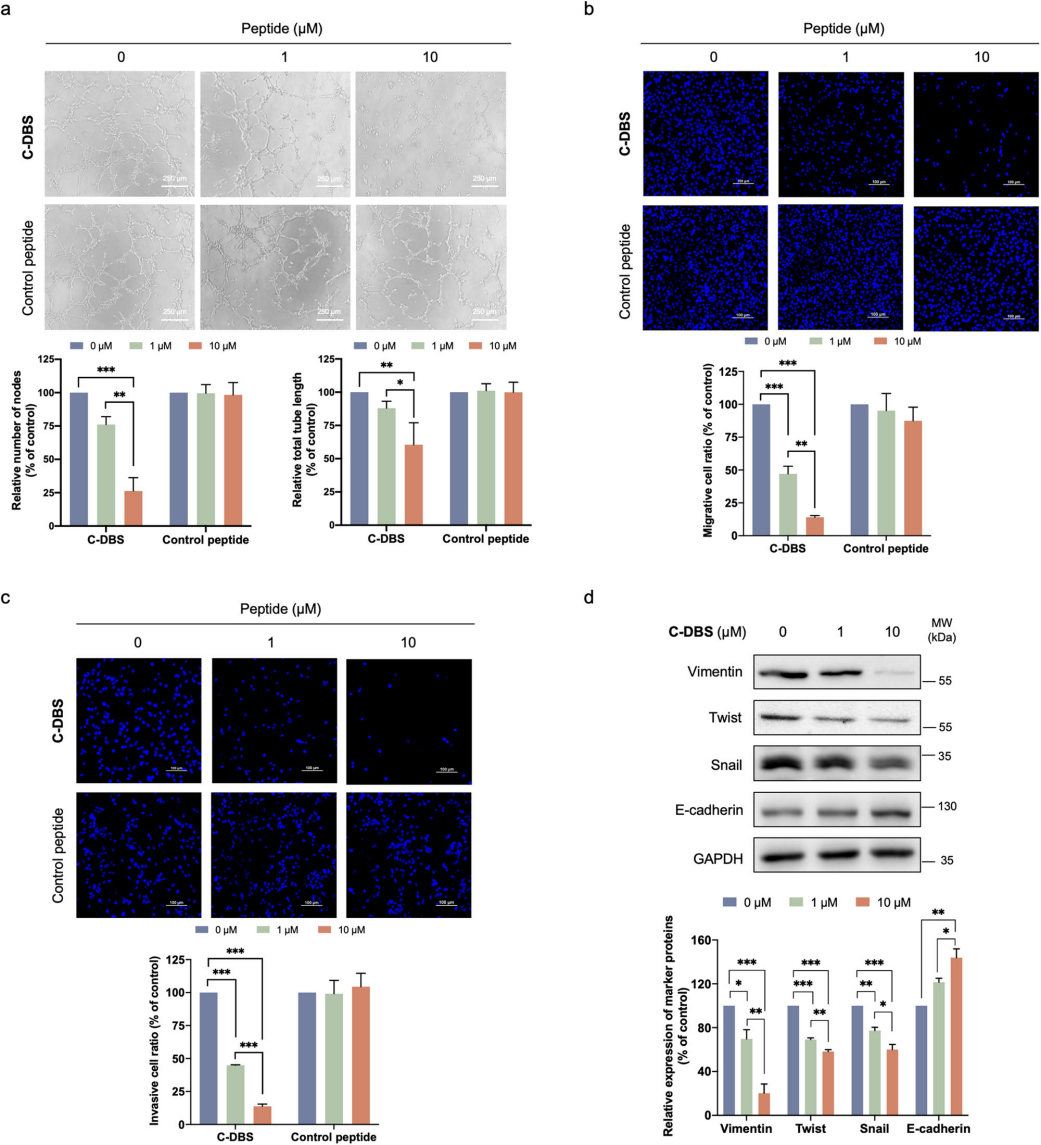
C-DBS suppresses angiogenesis, cell migration, and invasion. **a** C-DBS inhibited endothelial cell tube formation. Conditioned medium collected from A549 cells treated with C-DBS (top panel) or the control peptide (bottom panel) was added to HUVECs seeded in Matrigel-coated wells. The images of capillary-like tubes were captured at three random views in each well. The inhibitory effect of C-DBS on angiogenesis was determined by counting the number of tube nodes and measuring the length of the capillary-like tubes that formed using ImageJ. Scale bar: 250 μm. C-DBS inhibited the migration **b** and invasiveness **c** of A549 cells. A549 cells were resuspended in an FBS-free medium containing different concentrations of C-DBS (top panel) or the control peptide (bottom panel). Full medium containing 10% FBS was used as attractants in the lower chamber. Migrated or invaded cells were fixed and stained with Hoechst 33342 and photographed at five random views using a fluorescent microscope. The number of migrated or invaded cells was counted using ImageJ. **d** C-DBS altered the expression of EMT markers. A549 cell lysates with or without C-DBS treatment were subjected to Western blotting. EMT markers were detected using the corresponding antibodies. Band intensity quantification was performed using ImageJ. All data represent means ± SEM from three independent experiments. Statistical analysis was performed using one-way ANOVA. *p < 0.05; **p < 0.01; ***p < 0.001.

RNAi-mediated knockdown or inhibition of SRPK1 by using small molecule inhibitors or DBS1 has been demonstrated to shift the splicing of VEGF and subsequently inhibit angiogenesis^29,30,38^. To determine whether C-DBS can also inhibit angiogenesis, we performed an in vitro tube formation assay by using human umbilical vein endothelial cells (HUVECs). MTT assay was performed precedently to confirm C-DBS is noncytotoxic within the concentration range used (Supplementary Fig. S6). Conditioned medium (CM) collected from A549 cells treated with C-DBS or a reactive control peptide derived from the trans-activator of transcription (TAT) of HIV and the RRM2 region of SRSF1 (GRKKRRQR(K-aryl-SO_2_F)RPQSWQDLKDH) was added to HUVECs. Our results showed that C-DBS, but not the control peptide, reduced both the number of tube nodes and the total length of the capillary-like tubes in a dose-dependent manner (Fig. 7a and Supplementary Data 2). These results confirmed the potential of C-DBS as an inhibitor of angiogenesis.

**Fig. 8:**
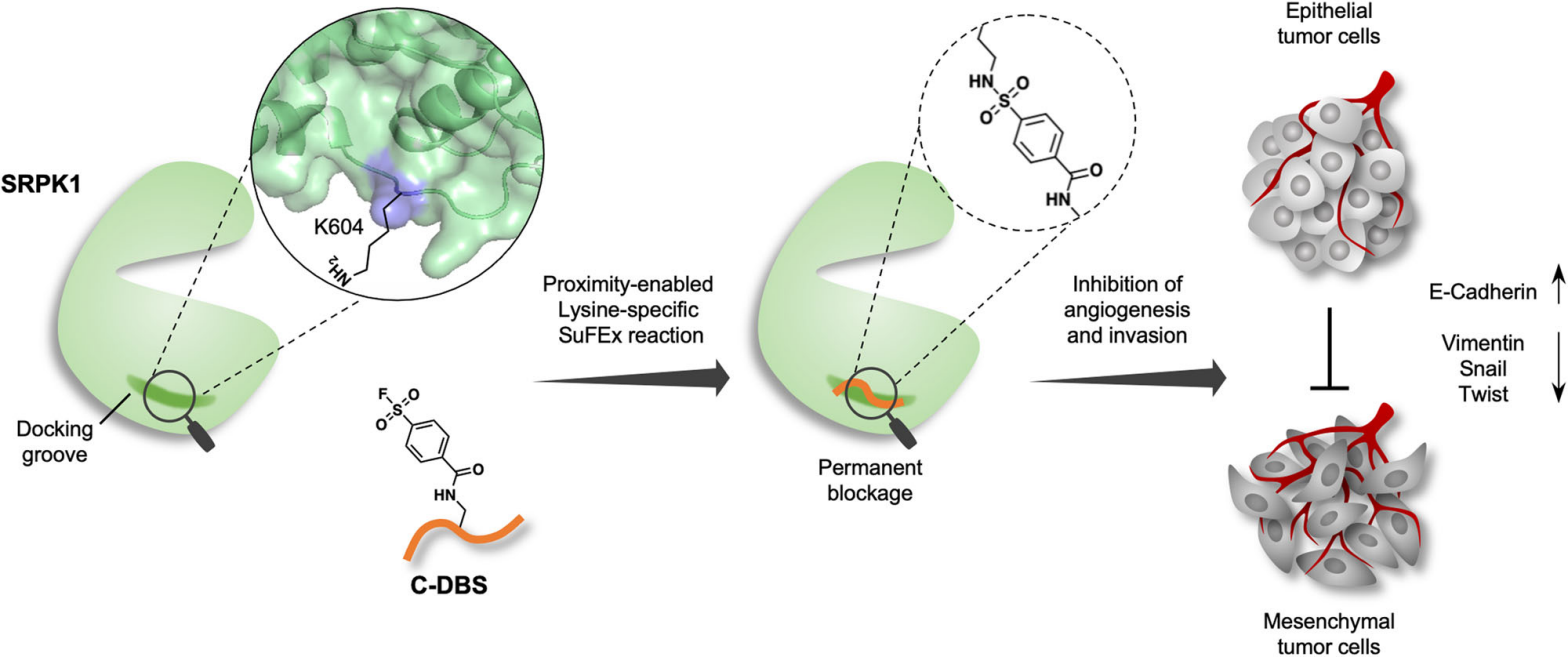
Schematic illustration of C-DBS combating angiogenesis and invasion. C-DBS permanently blocks the docking groove of SRPK1 via proximity-enabled SuFEx reaction with specific lysine, resulting in the anti-angiogenesis and anti-invasion effect by reversing the EMT of tumor cells.

Accumulating evidence indicates that the abnormal expression of SRPKs is correlated with the invasiveness and metastasis of cancer cells^39–43^. Therefore, we investigated the effect of C-DBS on cancer cell migration and invasion by performing Transwell assays. The results revealed that the migration of A549 cells was inhibited by 50% and 85% after the administration of 1 and 10 μM C-DBS, respectively. However, no changes in cell migration were observed when the reactive control peptide was administered (Fig. 7b and Supplementary Data 2). In addition, C-DBS significantly inhibited Matrigel invasion of A549 cells compared with those treated with the control peptide (Fig. 7c and Supplementary Data 2). We also evaluated the effects of C-DBS on the epithelial-to-mesenchymal transition (EMT). The expression of the mesenchymal markers, vimentin, twist, and snail was downregulated but that of the epithelial marker E-cadherin was upregulated (Fig. 7d and Supplementary Data 1, 2). These results suggested that C-DBS exhibited antimetastatic potential against NSCLC cells. To assess whether C-DBS is also effective in other cancers, we analyzed its effects on SR protein phosphorylation in the triple-negative breast cancer (TNBC) cell line MDA-MB-231 and repeated the Transwell assays. Similar to the results in A549 cells, the phosphorylation levels of SR proteins in the MDA-MB-231 cells were lower following treatment with C-DBS (Supplementary Fig. S7a). Moreover, C-DBS significantly inhibited the migration and invasion of TNBC cells dose-dependently (Supplementary Figs. S7b, c). Collectively, the findings demonstrated that C-DBS is an effective inhibitor of SRPKs with the potential to inhibit angiogenesis and metastasis (Fig. 8).

## Discussion

Compared with deep grooves usually found in the active sites of enzymes, PPI interfaces are characterized by their large and amorphous nature. Although small molecules can act as PPI inhibitors, they are often suboptimal for blocking shallow and flat PPIs due to limited interactions^44,45^. On the basis of the observed crystal structures of SRPK1 in complexes with different substrate peptides, we previously developed a peptide inhibitor DBS1 that targets and blocks PPI at the unique SRPK-specific substrate docking groove. However, DBS1 only exhibits moderate inhibitory activity, likely because the docking groove can mediate sliding of the bound substrate peptide. Although DBS1 was designed such that the loss of inhibitory activity due to sliding can be minimized, the design may not completely prevent its own sliding. Therefore, we modified DBS1 into a covalent inhibitor. Most covalent inhibitors target cysteine due to its high nucleophilicity^46^. However, its low abundance and susceptibility to oxidation limit cysteine’s utilization during the development of inhibitors for some protein targets. In fact, no cysteine is in proximity to the docking groove of SRPK. Thus, lysines at the docking groove were selected as targets in this study. Screening of multiple lysine-targeting warheads identified aryl-sulfonyl fluoride as the most favorable warhead for targeting K604 within the SRPK1 docking groove. The reaction between sulfonyl fluoride and lysine belongs to the sulfur–fluoride exchange (SuFEx) chemistry, which is recognized as a new generation of click chemistry following the typical azide–alkyne cycloaddition reaction^47–49^. SuFEx reactions have the advantages of high reactivity, specificity, reduction resistance, and thermodynamic stability^47^. Their effectiveness has been demonstrated in the development of inhibitors against diverse targets, including G protein-coupled receptors, carbonic anhydrase II, and transcription factor B-cell lymphoma^50^. We also found C-DBS to be stable in our assay because it continued to react with the kinase after 3 h. Notably, in our cell-based conjugation assays and cellular import studies, no apparent conjugation occurred with nonspecific proteins or membrane components, even when the incubation period was long. These findings suggest that the SuFEx reaction between C-DBS and SRPK is active and specific but mild enough to minimize nonspecific reactions. C-DBS binds to SRPK1 with an affinity at the nanomolar range and it exhibits adequate specificity to bring the warhead of C-DBS into proximity with the docking groove lysine, allowing the SuFEx reaction to proceed in cells. This indicates that C-DBS maintains a favorable balance between selectivity and reactivity, potentially overcoming concerns regarding off-target effects.

Unlike those of other therapeutic targets—such as G protein-coupled receptors, which have a wide range of druggable sites—the typical targeting site of kinases is the ATP-binding pocket due to the kinases’ conserved structures. However, this leads to potential polypharmacology and drug resistance caused by gatekeeper mutations^23,51^. Our strategy to target the unique docking groove of SRPKs, instead of the ATP-binding cleft, enables the selective inhibition of SRPKs over other kinases. Yet, the conservation of the targeted lysine in the docking grooves of SRPKs impairs C-DBS from differentiating the various members of the SRPK family. We previously identified that a key difference between the docking grooves of SRPK1 and SRPK2 lies at the center of the groove, where L568 in SRPK1 is replaced by H601 in SRPK2 (Supplementary Fig. S5a). This difference leads to differing processivities of the two closely related kinases^12^. Because histidine is a newly proposed potential nucleophilic residue for targeting^33,34^, a covalent inhibitor that can conjugate with the histidine residue of SRPK2 can be developed to achieve selectivity between the two kinases.

Pharmacological inhibition of SRPKs by ATP-competitive inhibitors has been reported to inhibit growth, proliferation, angiogenesis, migration, and invasion and reduce the CSC-like phenotype in cell or animal models. Our observation of the effective inhibition of angiogenesis, migration, and invasion of both NSCLC and TNBC cells by covalent inhibition of the docking groove confirms that this PPI is a target for anticancer therapeutic intervention. In addition to their roles in the regulation of SR protein, SRPKs have been implicated as cellular kinases that phosphorylate diverse viral proteins—including hepatitis B virus (HBV) core protein (HBc), herpes simplex virus protein ICP27, and the nucleocapsid proteins of both SARS-CoV and SARS-CoV2—to regulate their lifecycle^52–54^. Although the mechanisms underlying the interaction between most of these viral proteins and SRPKs remain unclear, our recent study revealed that the binding and phosphorylation of HBc by SRPK2 depend on the docking groove. This implies that our inhibitor not only has good potential for combating cancer but also for addressing viral infections such as HBV infection.

Peptide inhibitors have long been thought to suffer from the drawback of being prone to proteolytic degradation and the limitations of short plasma half-life and negligible oral bioavailability. However, given the advancements in synthetic strategies and medicinal chemistry techniques, candidates of peptide therapeutics have been improved to be more drug-like with favorable pharmaceutical properties^55^. For instance, the N-acetylation and C-amidation modifications adopted in this study have been proven to extend the stability and half-life of peptide inhibitors^56–58^.We and others have also shown that strategy like lipidation could significantly improve the stability and cellular uptake of peptides^56,59^. Additionally, DBS1 is predicted by PEP-FOLD4 and ColabFold to adopt alpha helical structure (Supplementary Fig. S8)^60,61^, making C-DBS a potential candidate for stapling modification for further stabilization. Combining these strategies with new formulation and delivery, C-DBS has great promise to be developed into a therapeutic agent for in vivo applications.

## Methods

### Cell lines used in this study

A549 (ATCC, CRM-CCL-185^TM^, human, male) and MDA-MB-231 (ATCC, HTB-26^TM^, human, female) were cultured in Roswell Park Memorial Institute (RPMI) 1640 Medium (Gibco) with 10% Fetal Bovine Serum (FBS) (Gibco) and 1% penicillin/streptomycin (Gibco). HeLa (ATCC, CRM-CCL-2^TM^, human, female) and HEK-293 (ATCC, CRL-1573^TM^, human, female) were cultured in Dulbecco’s Modified Eagle Medium (DMEM) (Gibco) with 10% FBS plus 1% penicillin/streptomycin. Cell passage was performed every 2–3 days regarding cell confluency. Human umbilical vein endothelial cells (HUVECs) (ATCC, CRL-1730^TM^) were cultured in Medium 199 (Gibco) with 20% heat-inactivated FBS and 1% penicillin/streptomycin, supplemented with 50 μg/mL endothelial cell growth supplement (ECGS) (Corning) and 100 μg/mL heparin (Sigma-Aldrich). The medium was replaced every other day. All the cells were cultured in a 37 ^o^C incubator with humidified 5% CO_2_.

### Cloning and expression of recombinant proteins

The mutation of SRPK1ΔNS3_K604A was based on the SRPK1ΔNS3 construct in the pET15b vector. The mutation was induced using site-direct mutagenesis with forward primer 5’-TTCAGGTCACCTTTTTTGGTGAAAAATTCC-3’ and reverse primer 5’-ACATATCACGAAGCTGGCACCTTGGG-3’. PCR products were purified using a gel extraction kit (Invitrogen), and template DNA was cleaved by DpnI (New England Biolabs). Products were transformed into DH5α strain *Escherichia coli* cells via heat shock. Sequencing was performed to verify the presence of the mutation.

Protein expression and purification of SRPK1 and SRSF1 were performed as described previously^13^. Briefly, recombinant proteins were expressed in BL21(DE3) pLysS strain *Escherichia coli* cells via heat shock. Large-scale cultures were grown in Terrific Broth (IBI Scientific) with 200 μg/mL ampicillin (Santa-Cruz) and 50 μg/mL chloramphenicol (Sigma-Aldrich) at 37 ^o^C. Protein expression was induced by 0.2 mM isopropylthiogalactoside (IPTG) (GOLD BIO) for 18–20 h at 16 ^o^C when O.D. reached 0.6–0.8. *E. coli* cells were pelleted and lysed via ultrasonication on ice. Recombinant SRPK1 and its mutant proteins were purified using an anion exchange column (Q-Sepharose) and a Ni-NTA affinity column (MACHEREY-NAGEL). Purified proteins were dialyzed into a buffer containing 20 mM MES pH 6.5, 300 mM NaCl, and 5% glycerol supplemented with 1 mM DTT. If necessary, the polyhistidine tag would be removed by thrombin (GE Healthcare) overnight at 25 ^o^C, and subsequent gel filtration purification would be performed using the Superdex 75 size exclusion column (GE Healthcare). Recombinant GST-tagged SRSF1 protein in pGEX-4T-2 vector was purified using anion exchange (Q-Sepharose) and GST columns (GenScript) and dialyzed into a buffer containing 20 mM Tris-HCl pH 7.5, 300 mM KCl, 10% glycerol, and 1 mM DTT. All the proteins were concentrated, flash-frozen, and stored at −80 ^o^C for further use.

### Peptide synthesis and characterization

All peptides were synthesized based on manual 9-fluorenylmenthoxycarbonyl (Fmoc) solid-phase peptide synthesis (SPPS) chemistry. Rink Amide-ChemMatrix resins with a loading capacity of 0.46 mmol/g were purchased from Biotage® (Uppsala). All Fmoc-protected amino acids were purchased from GL Biochem Ltd. Generally, in each coupling step, a five-fold excess of Fmoc-protected amino acid, 2-(1H-benzotriazol-1-yl)-1, 1, 3, 3-tetramethyluronium hexafluorophosphate (HBTU), *N*-hydroxy benzotriazole (HOBt), and *N, N*-diisopropylethylamine (DIPEA) with a ratio of 1: 1: 1: 2 in dimethylformamide (DMF) was added to the resin for peptide elongation. Then, the Fmoc group was removed in 20% piperidine in DMF (*v/v)* to expose the amine group for the next step of the coupling reaction. After finishing the coupling/deprotection cycles, the N terminus of the peptide was acetylated. To install modifications at a certain position of the peptide sequence, amino acids with specific side-chain protections were used. For DBS1-1 and DBS1-3, 4-methyltrityl (Mtt)-protected lysine residue was used at the 9th position. After acetylation, the Mtt group was removed by trifluoroacetic acid (TFA)/deionized water/triisopropylsilane (TIPS) (3/94/3) at room temperature and the modifications were introduced by reacting the peptidyl-resin with a five-fold excess of 4-fluorosulfonylbenzoic acid (Sigma-Aldrich) and 1,5-difluoro-2,4-dinitrobenzene (Sigma-Aldrich), respectively. For DBS1-2, DBS1-4, and DBS1-5, allyl (All)-protected glutamic acid residue was used at the 9th position. After acetylation, the All group was removed by tetrakis(triphenylphosphine)palladium(0) [Pd(PPh_3_)_4_] (0.2 equiv.) (TCI) and phenylsilane (PhSiH_3_) (Macklin) in dichloromethane (DCM). Then, the resin was washed with sodium (diethylcarbamothioyl)sulfanide (10 mg/mL in DMF) (J&K Scientific). 4-fluorophenyl, 4-nitrophenyl, and 4-phenyl groups were introduced by five-fold excess of 4-fluorophenol, 4-nitrophenol, and phenyl (J&K Scientific). The synthesis of DBS1-1.2, DBS1-1.15, DBS1-1b, and DBS1-1p was similar to that of DBS1-1, except the modification was installed at the 2nd or 15th positions, the Mtt-protected lysine was replaced by diaminobutyric acid (Dab) or diaminopropionic acid (Dap). Carboxyfluorescein (FAM)-label C-DBS was synthesized by introducing a cysteine to the N-terminus after Fmoc deprotection, allowing the reaction with 6-FAM maleimide in 1 M citric acid solution. After finishing modifications, the peptides were cleaved from the resin in a cleavage cocktail (TFA/DCM/TIPS (95:2.5:2.5)) at room temperature. The crude peptide was obtained by precipitation by adding cold diethyl and purified by RP-HPLC (Prominence LC 20-A, Shimadzu, Kyoto) with a Vydac 218TP C18 LC column (10 µm, 250 mm × 10 mm, 300 Å) at a flow rate of 3 mL/min and confirmed by MALDI-TOF MS analysis (Bruker Daltonics).

### Protein-peptide adduction assay

Peptides (DBS1-1, DBS1-2, DBS1-3, DBS1-4, DBS1-5, DBS1-1.2, DBS1-1.15, DBS1-1b, and DBS1-1p) at a concentration of 8 μM was added to 2 μM SRPK1ΔNS3 in a conjugation buffer (50 mM HEPES pH 7.5, 150 mM NaCl, 5% glycerol, and 1 mM benzamidine) for indicated time periods. The reactions were quenched by adding SDS loading buffer and boiling. Obtained mixtures were then resolved by SDS-PAGE and stained with Coomassie Blue. Conjugation assays with SRPK2ΔS1, BSA, GST, CLK1(148-484), SRPK1ΔNS3_DM, and SRPK1ΔNS3_K604A with C-DBS were performed in the same buffer condition.

### GST pull-down assay

GST-SRSF1 (500 nM) was immobilized by glutathione resin (Genscript) in a pull-down buffer (300 mM NaCl, 20 mM HEPES pH 7.5, 5% glycerol, 0.5% Triton X-100, 1 mM DTT, 1 mM benzamidine) for at least 4 h and washed three times to remove the unbound proteins. SRPK1ΔNS3 (1 μM) was incubated with indicated concentrations of C-DBS at room temperature for one hour, and the mixtures were then added to the resins and allowed to incubate for 20 min at 4 ^o^C. Afterward, the resins were washed with pull-down buffer (four times, 200 μL each), and proteins were eluted with SDS loading buffer, followed by boiling for 5 min. Samples were then resolved by SDS-PAGE and visualized by Coomassie Blue staining.

### Kinase activity assay

SRPK1ΔNS3, SRPK2ΔS1, CLK1, and Akt (Abcam) at a concentration of 10 nM were incubated with different concentrations of C-DBS in a kinase reaction buffer (100 mM NaCl, 50 mM Tris-HCl pH 7.5, 10 mM MgCl_2,_ and 5 mg/mL BSA) at room temperature for 1 h prior the addition of 500 nM GST-SRSF1. Reactions were initiated by adding 50 μM unlabeled ATP mixed with 0.5 μCi of [^32^P]ATP (PerkinElmer) and quenched with SDS loading buffer and boiling. The mixtures were resolved by 10% SDS-PAGE. Gels were dried using a gel dryer (Savant SG2000 Digital Slab Gel Dryer). The radioactive bands were exposed by autoradiography using X-ray films (Fujifilm) and photographed by BioRad ChemiDoc MP System. Band intensities were quantified by ImageJ software, and IC_50_ was calculated by GraphPad Prism software.

### Fluorescence polarization assay

FAM-C-DBS was used as the tracer titrated with SRPK1ΔNS3 or SRPK1ΔNS3_DM proteins. Briefly, 200 nM FAM-C-DBS was added into a 96-well black plate (GREINER, Cat. No. 655209). Serially diluted proteins were added into each well at an equal volume. The peptide and the protein were incubated at room temperature for 1 h before processing for the fluorescence reading by a microplate reader (Tecan Spark 10 M Microplate Reader). The *K*_d_ values for the interaction of C-DBS with SRPK1ΔNS3 or SRPK1ΔNS3_DM were calculated using GraphPad Prism software.

### LC-MS/MS analysis of the C-DBS-SRPK1 adduct

The adduct band on SDS-PAGE gel in the conjugation assay was cut and digested with trypsin (Promega) and Glu-C (Promega) at 37 ^o^C overnight. The enzymatic digestion was quenched through the addition of formic acid (10%), and the peptides were collected and vacuum-dried prior to mass analysis. MS data were acquired on an Orbitrap Fusion mass spectrometer (Thermo Scientific) equipped with EASY-nLC 1200 system (Thermo Scientific) and EASY-Spray HPLC column (75 μm I.D. × 150 mm, 3 μm, 100 Å) and ion source (Thermo Scientific). The chromatographic separation was performed using 0.1% formic acid in water as mobile phase A and 0.1% formic acid in 80% acetonitrile as mobile phase B operated at the flow rate of 300 nL/min. The MS/MS analyses were carried out with the collision-induced dissociation (CID) mode with the collision energy of 35%. Acquired MS raw data were converted as mgf format by msConvert (version 3.0.18165, ProteoWizard), then analyzed using MassMatrix for MS/MS ion search of cross-linked peptides.

### Cell viability assay

Cells (6 × 10^3^ per well) were seeded into a 96-well plate. After overnight incubation, cells were treated with different concentrations of C-DBS for 24 h. The viability of the cells was determined using 3-(4,5-dimethylthiazol-2-yl)-2,5-diphenyltetrazolium bromide (MTT) (Roche). Absorption at 570 nm was measured using a microplate reader (BMG, CLARIOstar) according to the manufacturer’s instructions.

### Cellular uptake and endo/lysosomal escape

A549 cells (20 × 10^4^ per well) were seeded into confocal dishes (SPL Life Sciences) and incubated for adhesion overnight at 37 ^o^C in 5% CO_2_. 6-FAM-labeled C-DSB or control peptide was diluted to Opti-MEM medium (Gibco) and added to the cells for 24-h incubation. C-DBS was washed with PBS containing 20 U/mL heparin (Sigma-Aldrich) and stained with 40 μM Hoechst 33342 (Life Technologies) and 5 μg/mL Wheat Germ Agglutinin Alexa Fluor 647 Conjugate (Life Technologies) for 20 min. After removing excess dye, cell images were captured by the Leica TCS SP8 Confocal Microscope System. To detect the endo/lysosomal escape of C-DBS, 50 nM Lysotracker Red (Life Technologies) was used to stain the endo/lysosomes. Fluorescence intensity profiles of the region of interest (ROI) were analyzed using ImageJ software.

### Preparation for mAb104 antibody

Hybridoma cells mAb104 (ATCC, CRL-2067^TM^) were cultured in Iscove’s Modified Dulbecco’s Medium (IMDM) (ATCC) plus 20% FBS and 1% penicillin/streptomycin. Cells were passaged every 2–3 days. The culture medium was collected and spun at 5000 RPM for 15 min before the supernatant was directly used for western blotting.

### Inhibitor treatment and western blotting

A549 cells (10 × 10^4^ per well) were seeded into a 12-well plate, starved with Opti-MEM (Gibco) for 24 h, and treated with C-DBS in RPMI 1640 medium. At the time of harvest, the cells were washed with cold PBS and then lysed on ice with RIPA buffer (Cell Signaling Technology) supplemented with protease inhibitor cocktail (MedChemExpress), phosphatase inhibitor cocktail (Sigma), 10 mM sodium deoxycholate (Sigma-Aldrich), 10 mM sodium pyrophosphate decahydrate (Sigma-Aldrich) and 10 mM sodium fluoride (Sigma-Aldrich). Cell lysates were centrifuged at 4 ^o^C for 15 min to remove cell debris. The supernatants were collected, heated, and resolved by SDS-PAGE. The separated proteins were transferred to nitrocellulose membranes (0.22 μm, Bio-Rad) and blocked with 5% BSA (AMRESCO) or 5% non-fat milk in TBST buffer (20 mM Tris-HCl, pH 7.6, 150 mM NaCl, 0.1% Tween 20). Membranes were incubated with primary antibody mAb104 (ATCC, Cat. No. CRL-2067), β-tubulin (1:2000, Genscript, Cat. No. A01717-40), vimentin (1:500, Abcam, Cat. No. AB92547), E-cadherin (1:500, Abcam, Cat. No. AB40772), GAPDH (1:1000, Abcam, Cat. No. AB8245), snail (1:300, Life Technologies, Cat. No. MA514801), twist (1:150, Santa Cruz Biotechnology, Cat. No. SC-81417) at 4 ^o^C overnight and then washed three times with TBST. Mouse IgGκ BP-HRP (1:10000, Santa Cruz Biotechnology, Cat. No. SC-516102) and Goat anti-rabbit IgG (H + L) HRP (1:10000, Life Technologies, Cat. No. G21234) were used as secondary antibodies. Membranes were then washed three times with TBST and visualized using enhanced chemiluminescence (Cytiva) substrate or ultra-high sensitivity ECL kit (MedChemExpress).

### Tube formation assay

A549 Cells (10 × 10^4^ per well) were seeded into a 12-well plate and starved with Opti-MEM for 24 h. C-DBS or control peptide was diluted in Opti-MEM and added to the cells. After a 16-h incubation, the culture medium in each well was collected and spun at 900 RPM. The obtained supernatant was used as the conditioned medium (CM). Before harvesting the HUVECs, 50 μM Matrigel (Corning) was added into the 96-well plate and incubated at 37 ^o^C for 1 h to polymerize. HUVECs were then trypsinized and seeded (1 × 10^4^ cells per well) into the Matrigel-coated 96-well plate, with the addition of 100 μL of CM to the corresponding wells. After incubation for 4–6 h, pictures of the formed tubes were captured using a microscope (NIKON, ECLIPSE TE300) at three different views in each well. The tube nodes and total length were measured with ImageJ software.

### Cell migration and invasion assays

A549 or MDA-MB-231 Cells (4 × 10^4^ per well) were seeded into 24-well plates and starved for 24 h. Cells were then trypsinized, washed twice with PBS, resuspended in an FBS-free RPMI 1640 medium containing different concentrations of C-DBS, and seeded into 8 μm transwell inserts (SPL Life Sciences). RPMI 1640 medium containing 10% FBS was added into the lower chamber as an attractant. After incubation for 16 or 24 h, migrated cells were fixed with 4% formaldehyde solution (Sigma-Aldrich) and stained with 40 μM Hoechst 33342 for 15 min. The cells in the upper chamber were removed with cotton swabs. Inserts were rinsed in PBS to remove excess dye and unfixed cells. Images were taken using Carl Zeiss PALM Inverted Microscope at five random regions. Migrated cells were counted by ImageJ software. The invasion assay was performed similarly to the migration assay except that the transwell inserts were pre-coated with 0.5 mg/mL Matrigel matrix (Corning).

### Quantification and statistical analysis

The intensities of protein bands and radioactive bands were quantified using ImageJ software. The number of tube nodes, tube total length, migrated and invaded cells were measured with ImageJ software. Data were presented as means ± SEM from three replicates. One-way ANOVA was used to determine the statistical significance of the differences in multiple groups. The differences were considered to be statistically significant when p-values were < 0.05. *p < 0.05; **p < 0.01; ***p < 0.001.

### Reporting summary

Further information on research design is available in the Nature Portfolio Reporting Summary linked to this article.

### Supplementary information

Supplementary Information

Description of Additional Supplementary Files

Supplementary Data 1

Supplementary Data 2

Reporting Summary

## Data Availability

All data supporting the findings of this study are available within the paper and its Supplementary Information file. Unprocessed gels for the figures in the main manuscript is available in Supplementary Data 1. Numerical source data for graphs in the main manuscript is available in Supplementary Data 2. All other data or sources are available from the corresponding author upon reasonable request.
