# Supplementary material for: Design of a covalent protein-protein interaction inhibitor of SRPKs to suppress angiogenesis and invasion of cancer cells
Source: Commun Chem. 2024 Jun 27;7:144. doi: 10.1038/s42004-024-01230-2 (PMC11211491; doi:10.1038/s42004-024-01230-2)
Supplement: Supplementary file 1 — Supplementary Information [file 42004_2024_1230_MOESM1_ESM.pdf]

Design of a Covalent Protein-Protein Interaction Inhibitor of SRPKs to Suppress Angiogenesis and Invasion of Cancer Cells

Gongli Cai, Yishu Bao, Qingyun Li, Pang-Hung Hsu, Jiang Xia, and Jacky Chi Ki Ngo

Supplementary Figures

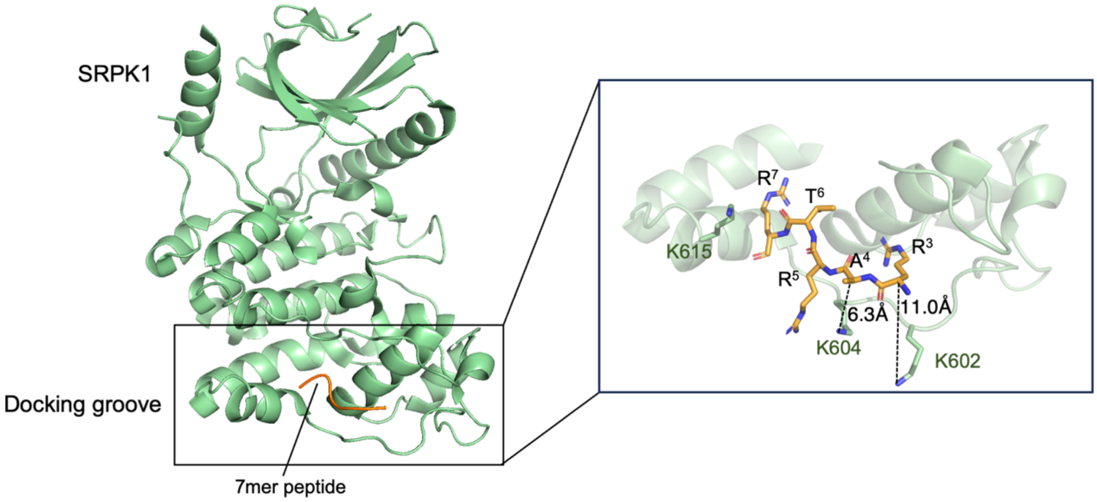

Figure S1. SRPK-specific docking groove.

The crystal structure of SRPK1 in complex with a 7mer peptide (PDB ID: 7DD1). The peptide (orange) binds at the SRPK-specific docking groove. Inset: the lysine residues K602, K604 and K615 within the docking groove that serves as potential sites for proximity-enabled conjugation reaction are shown. The sidechain of E2 is not modeled due to lack of electron density. Based on the location of R3, E2 is expected to locate closely to K604 and K602. The distances between the amino groups of both lysines and the closest Cα of the 7mer are indicated.

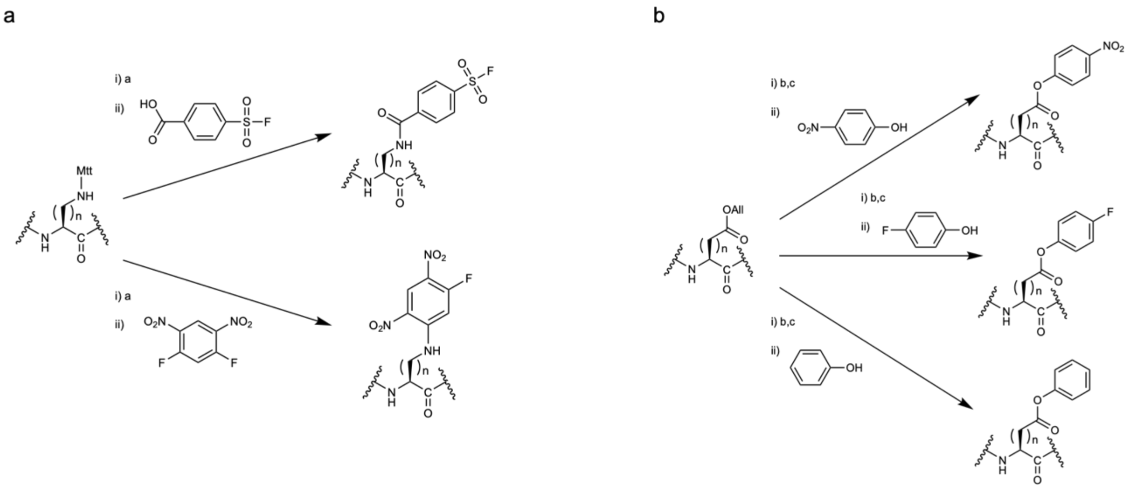

Figure S2. Synthesis of modified DBS1.

(a) Incorporation of aryl-sulfonyl fluoride and 1-fluoro-2,4-dinitrobenzene (FDNB) groups. (a) trifluoroacetic acid (TFA)/deionized water/triisopropylsilane (TIPS) (3/94/3), rt.

(b) Incorporation of 4-fluorophenyl, 4-nitrophenyl, and phenyl groups. (b) tetrakis(triphenylphosphine)palladium(0) [$Pd(PPh_3)_4$] and phenylsilane ($PhSiH_3$), DCM, rt; (c) sodium (diethylcarbamothioyl)sulfanide, DMF (10mg/mL), rt.

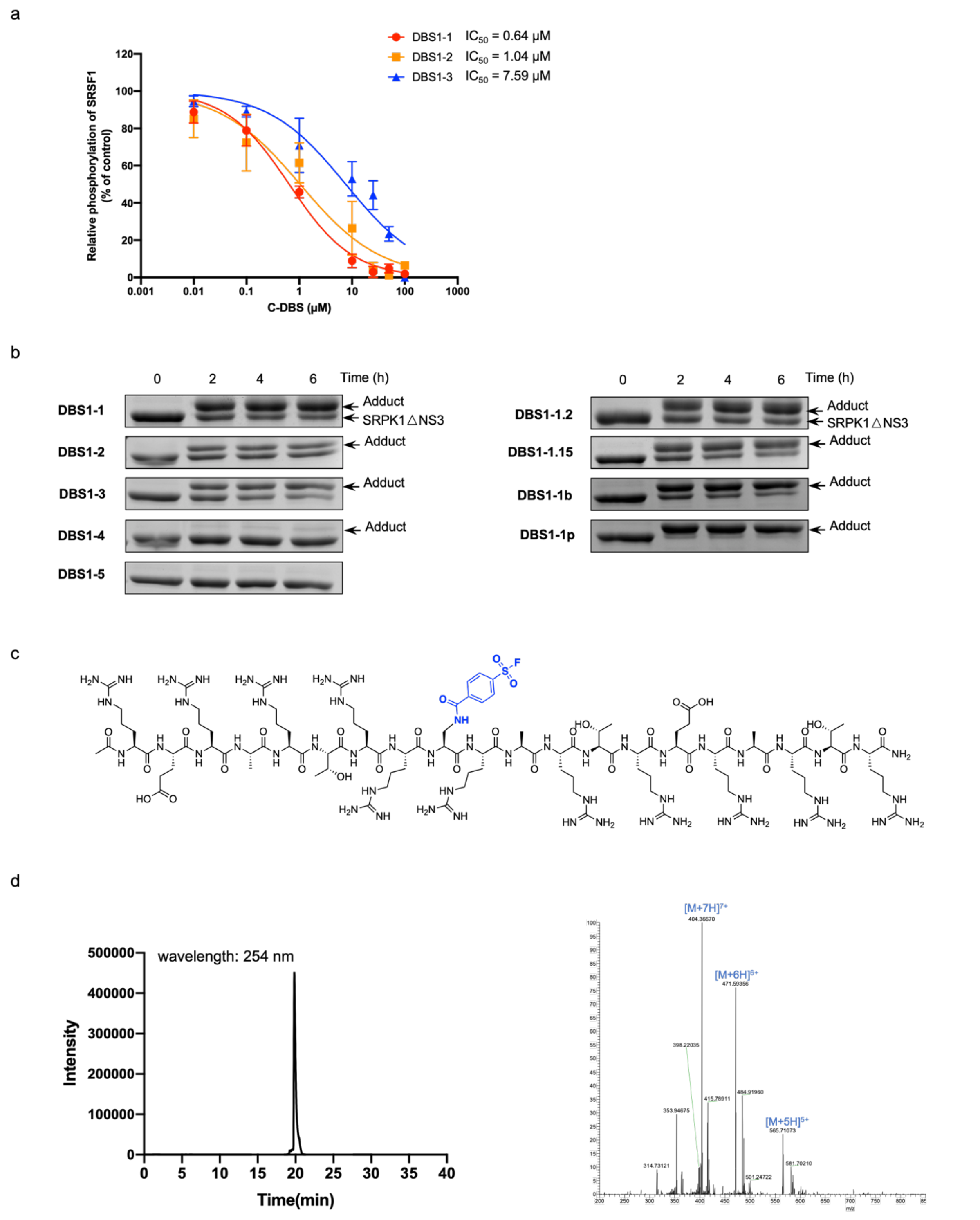

Figure S3. Development of **C-DBS**.

(a) Comparison of IC_{50} values of **DBS1-1**, **DBS1-2**, and **DBS1-3**. SRSF1 was phosphorylated by SRPK1△NS3 in the presence of different concentrations of the modified peptides using radioactive kinase activity assays. Radiolabelled phosphor-SRSF1 bands were quantified by ImageJ. **DBS1-1** showed the best inhibitory effect

among three reactive peptides with an IC_{50} of 640 nM. Data represent means ± SEM from three independent experiments.

(b) Adduct formation assays of different modified peptides with SRPK1 for extended time periods. The reaction was performed in a concentration of 1:4 (protein: peptide) at indicated time periods. Samples were resolved by SDS-PAGE and visualized by Coomassie Blue. Only **DBS1-1p** fully consumed SRPK1△NS3.

(c) Chemical structure of **C-DBS** incorporated with an aryl-sulfonyl fluoride group in the middle. **C-DBS** was N-terminal acetylated and C-terminus amidated.

(d) HPLC (left panel) and MALDI-TOF MS (right panel) analysis of **C-DBS**. Fragment peaks of $[M+5H]^{5+}$, $[M+6H]^{6+}$, $[M+7H]^{7+}$ were found in the mass spectrum.

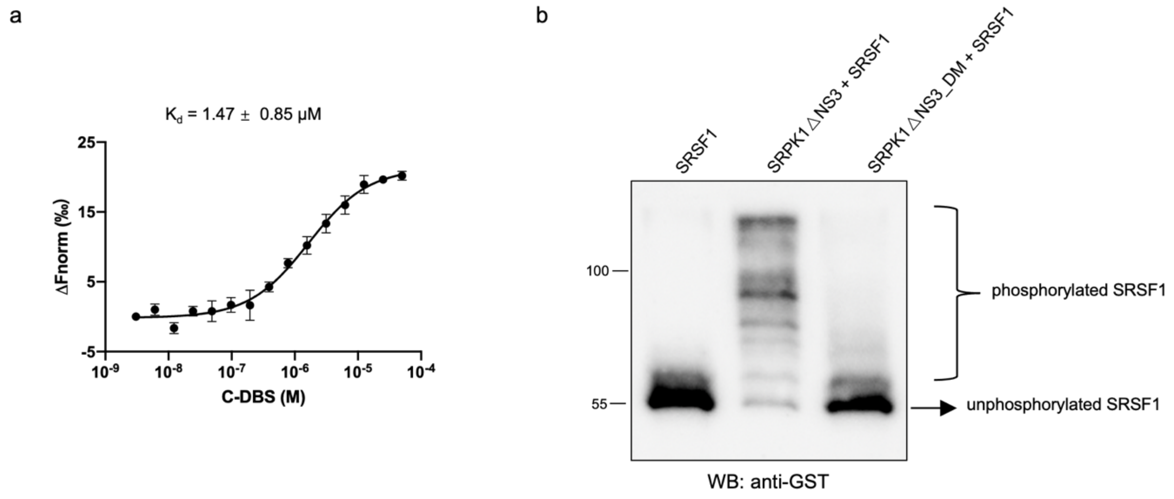

Figure S4. SRPK1 docking groove residues are important for the phosphorylation of substrate.

(a) The binding affinity between SRPK1△NS3 and C-DBS was measured using MST. A constant concentration (50 nM) of His-SRPK1△NS3 was titrated with varying concentrations of C-DBS, yielding a K_d value of 1.5 μM. Data represent means ± SEM from three independent experiments.

(b) SRPK1∆NS3 or SRPK1∆NS3_DM was used to phosphorylate GST-SRSF1. The activity assay was initiated by adding ATP and quenched by adding SDS loading buffer. Samples were resolved by Phos-tag SDS-PAGE and probed with anti-GST antibody. Nearly all SRSF1 was phosphorylated by SRPK1∆NS3 and resulted in shifted bands. In contrast, most SRSF1 remained unphosphorylated in the presence of SRPK1∆NS3_DM.

a

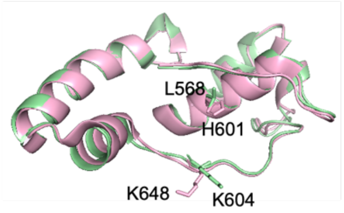

b

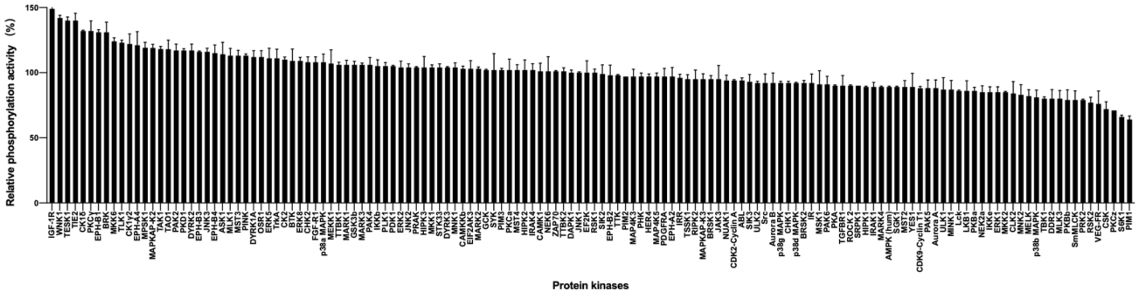

Figure S5. **C-DBS** specifically targets SRPKs

(a) Structure alignment of the docking grooves of SRPK1 (green) and SRPK2 (pink). The two structures were superimposed using the α carbons of the kinases. The docking grooves of the two kinases are highly conserved, except that L568 in SRPK1 is replaced by H601 in SRPK2. K648 of SRPK2 corresponds to K604 of SRPK1.

(b) **C-DBS** shows no inhibitory effect on other kinases. Related to Figure 4.

Screening of the inhibitory effect of **C-DBS** (500 nM) against 140 protein kinases was performed by MRC PPU International Centre for Kinases Profiling, University of Dundee. **C-DBS** showed no obvious inhibition toward the kinases. Experiments were conducted in duplicate, and data were presented as means ± SEM. Abbreviations and assay conditions used for each kinase are defined at https://www.kinase-screen.mrc.ac.uk.

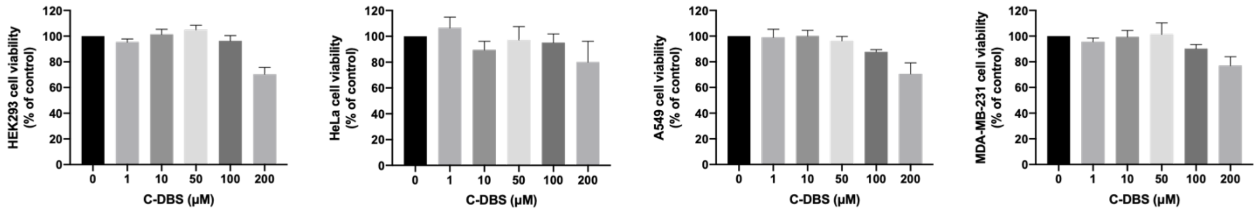

Figure S6. **C-DBS** is non-cytotoxic.

Cell viability in several cell lines, including A549, MDA-MB-231, HeLa, and HEK-293 after **C-DBS** administration. Cells were treated with increasing concentrations of **C-DBS** for 24 hours before adding MTT. The absorbance at 570 nm was determined according to the manufacturer's instructions. **C-DBS** showed no cytotoxicity to these cell lines at concentrations up to 100 μM. Data represent means ± SEM from three independent experiments.

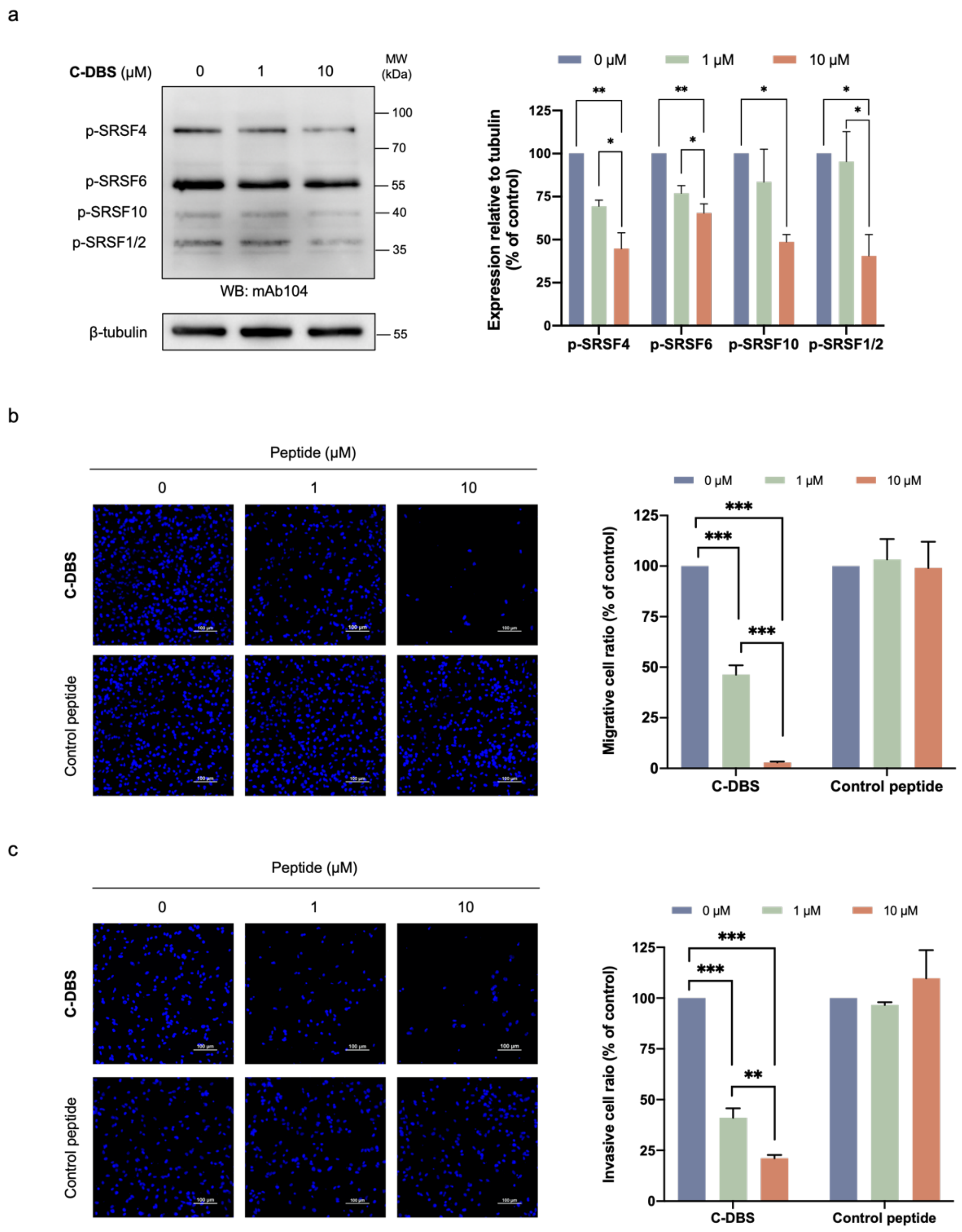

Figure

Figure S7. **C-DBS** inhibits the migration and invasion of MDA-MB-231 breast cancer cells.

(a) **C-DBS** down-regulated phosphor-SR proteins in MDA-MB-231 cells in a dose-dependent manner. MDA-MB-231 cells were treated with indicated concentrations of **C-DBS** and lysed with RIPA buffer supplemented with protease and phosphatase inhibitors. Total cell lysate was subjected to western blotting and phosphorylated SR proteins were probed with mAb104. The remaining p-SR levels versus β-tubulin were quantified using ImageJ. Data represent means ± SEM from three independent

experiments. Statistical analysis was performed using one-way ANOVA. $^*p < 0.05$; $^{**}p < 0.01$.

(b-c) **C-DBS** inhibited the migration (b) and the invasion (c) of MDA-MB-231 cells in a dose-dependent manner. MDA-MB-231 cells were resuspended in an FBS-free RPMI-1640 medium containing different concentrations of **C-DBS** (top panel) or the control peptide (bottom panel). Full medium containing 10% FBS was used as attractants in the lower chamber. Migrated or invaded cells were fixed and stained with Hoechst 33342 and photographed at five random views by fluorescent microscope. The number of migrated or invaded cells was counted using ImageJ. Data represent means ± SEM from three independent experiments. Scale bar: 100 μm. Statistical analysis was performed using one-way ANOVA. $^*p < 0.05$; $^{**}p < 0.01$; $^{***}p < 0.001$.

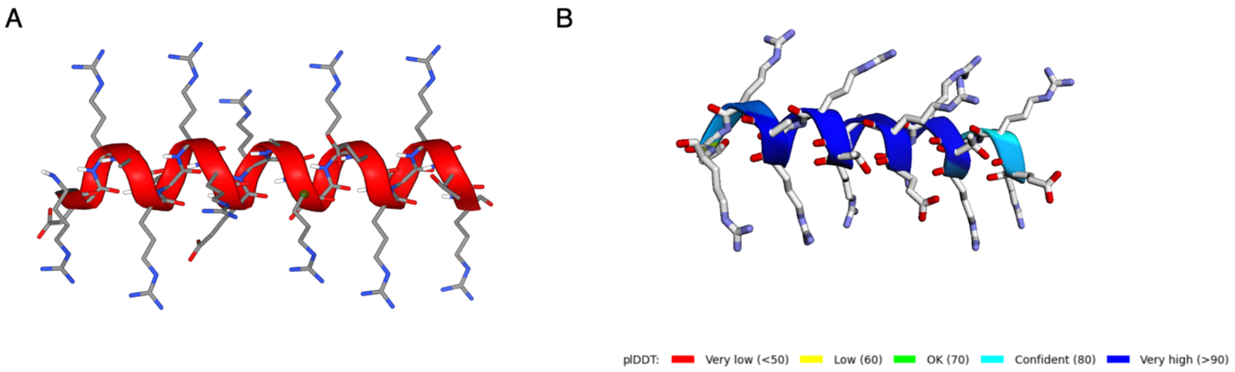

Figure S8. Predicted structure of DBS1.

DBS1 is predicted to adopt alpha helical structure by both PEP-FOLD4 and ColabFold – a user-friendly derivative of AlphaFold2. (a) Direct output of DBS1 as predicted by the PEP-FOLD4 server (https://bioserv.rpbs.univ-paris-diderot.fr/services/PEP-FOLD4/). (b)The structure predicted by ColabFold is coloured by the pLDDT scores of AlphaFold2.
