# Supplementary material for: Design of a covalent protein-protein interaction inhibitor of SRPKs to suppress angiogenesis and invasion of cancer cells
Source: Commun Chem. 2024 Jun 27;7:144. doi: 10.1038/s42004-024-01230-2 (PMC11211491; doi:10.1038/s42004-024-01230-2)
Supplement: Supplementary file 2 — Description of Additional Supplementary Files [file 42004_2024_1230_MOESM2_ESM.pdf]

Description of Additional Supplementary Files

File name: Supplementary Data 1

Description: Unprocessed gels for figures

File name: Supplementary Data 2

Description: Numerical source data for graphs
