# Supplementary material for: Design of a covalent protein-protein interaction inhibitor of SRPKs to suppress angiogenesis and invasion of cancer cells
Source: Commun Chem. 2024 Jun 27;7:144. doi: 10.1038/s42004-024-01230-2 (PMC11211491; doi:10.1038/s42004-024-01230-2)

Supplementary data for Figure 1. Conjugation of DBS1-1

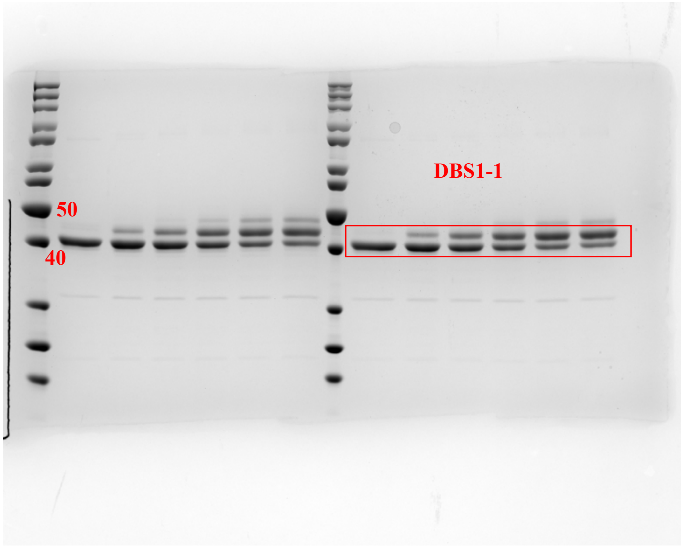

Supplementary data for Figure 1. Conjugation of DBS1-2 and DBS1-3

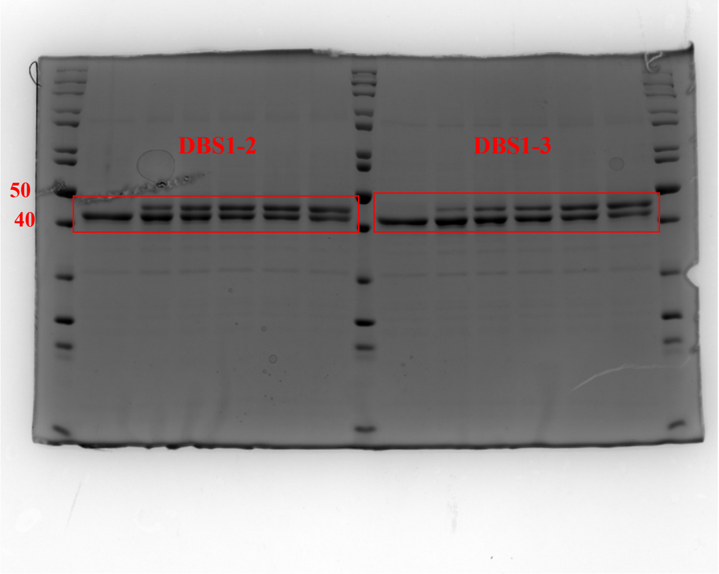

Supplementary data for Figure 1. Conjugation of DBS1-4

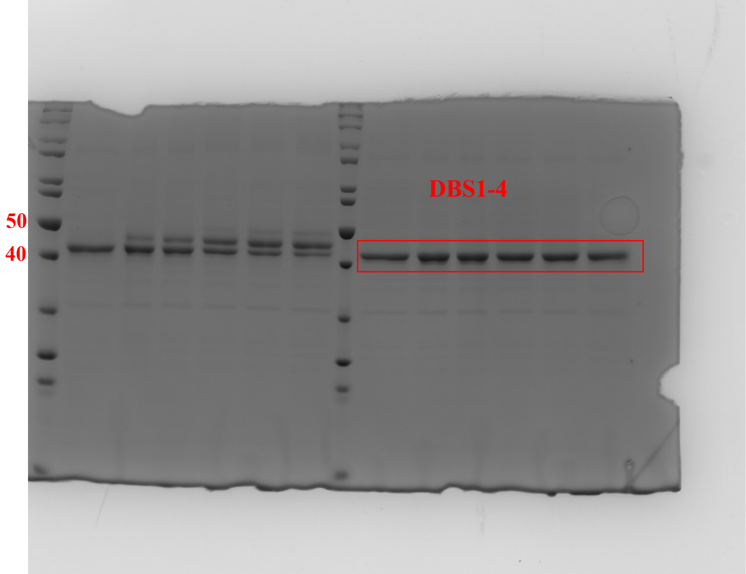

Supplementary data for Figure 1. Conjugation of DBS1-5

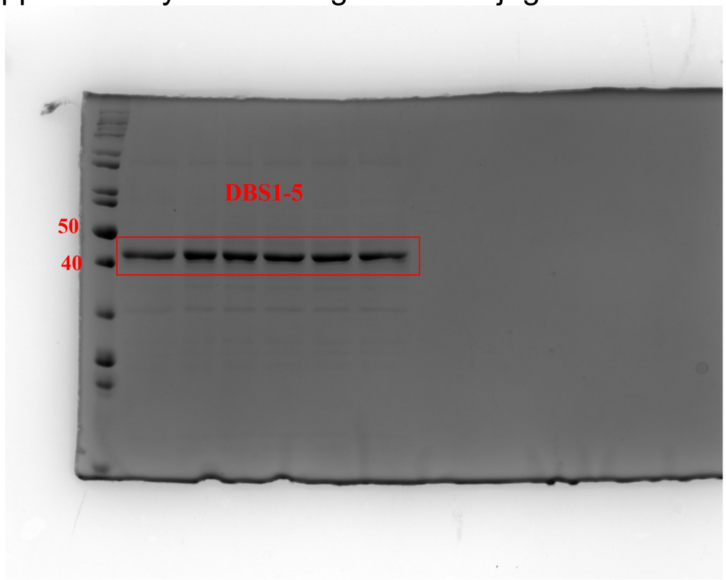

Supplementary data for Figure 1. Conjugation of DBS1-1.2

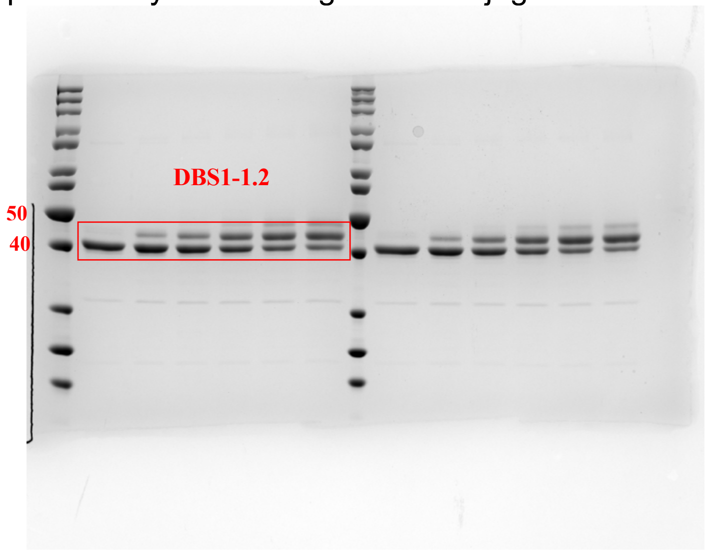

Supplementary data for Figure 1. Conjugation of DBS1-1.15

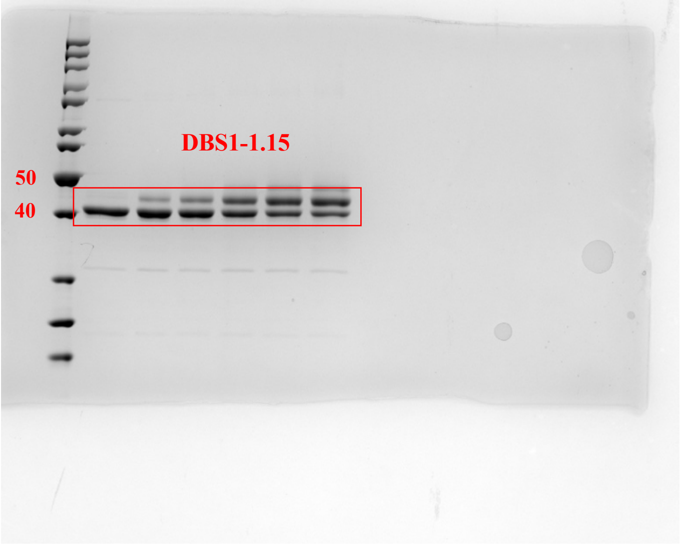

Supplementary data for Figure 1. Conjugation of DBS1-1b

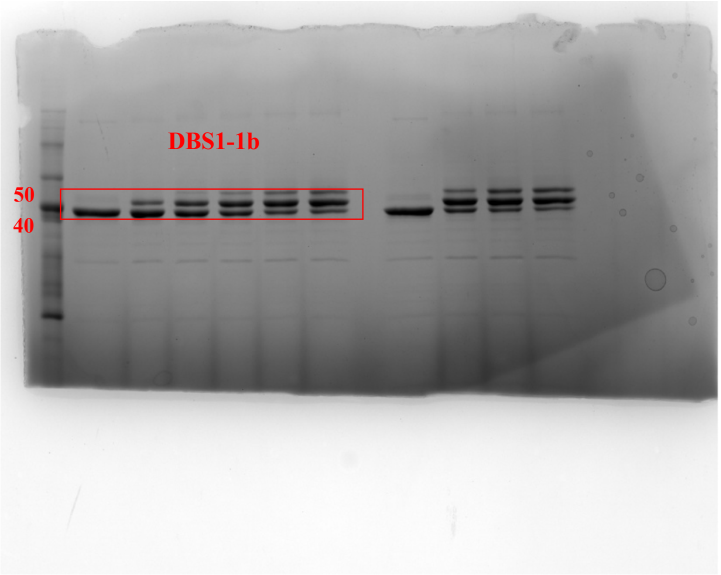

Supplementary data for Figure 1. Conjugation of DBS1-1p

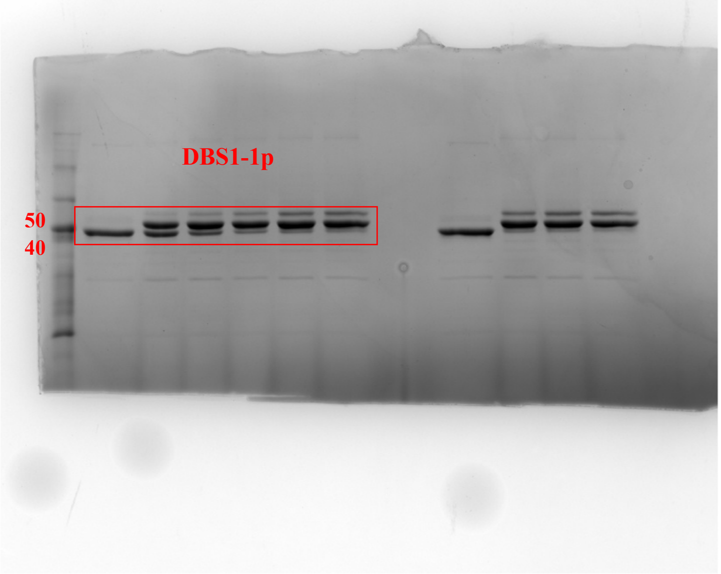

Supplementary data for Figure 1. Kinase activity of DBS1-1

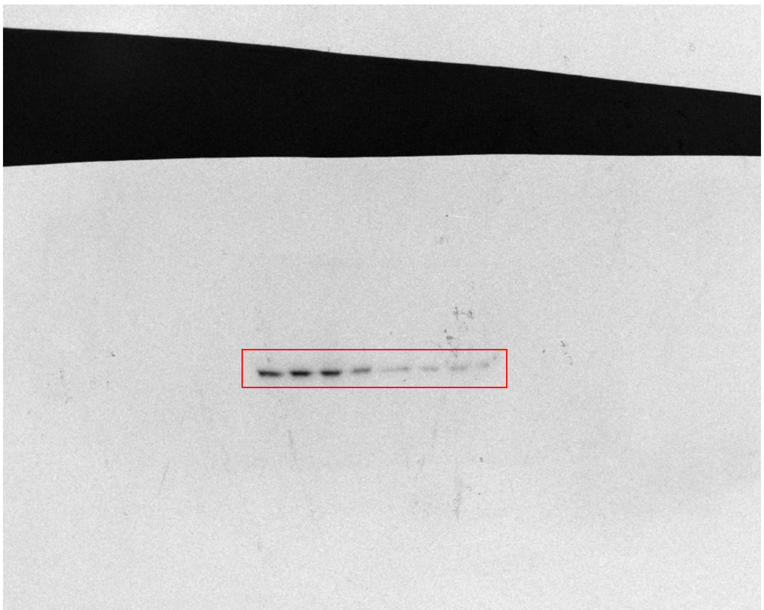

Supplementary data for Figure 1. Kinase activity of DBS1-2

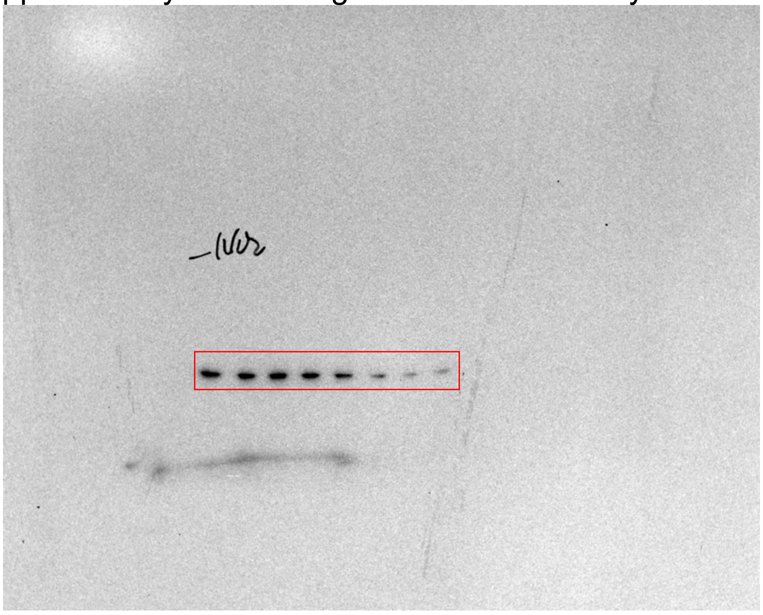

Supplementary data for Figure 1. Kinase activity of DBS1-3

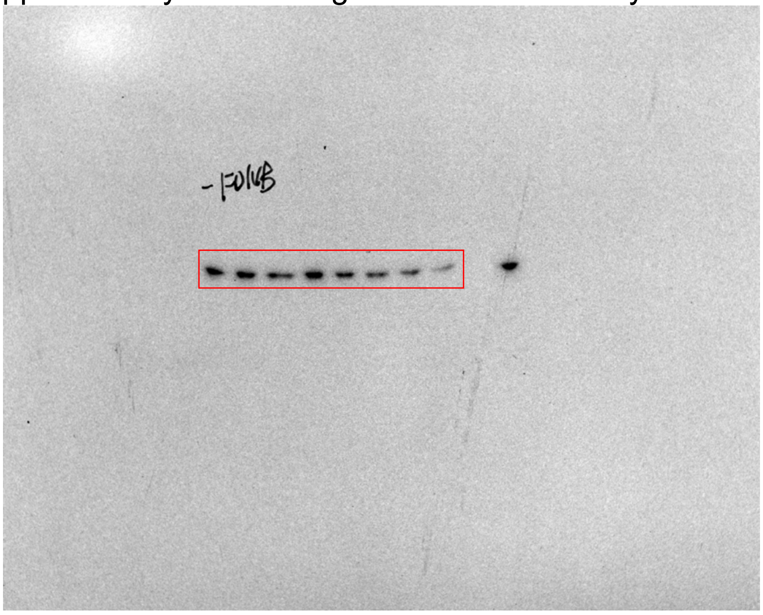

Supplementary data for Figure 1. Kinase activity of DBS1-1.2

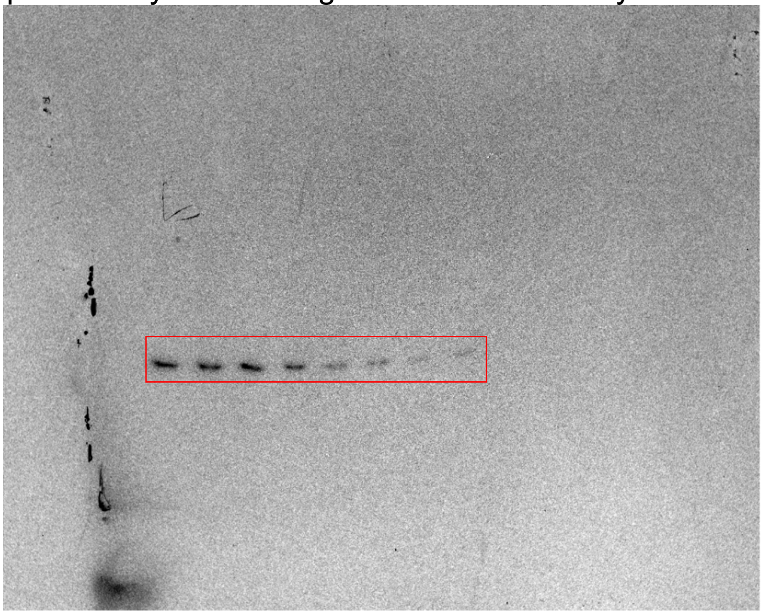

Supplementary data for Figure 1. Kinase activity of DBS1-1.15

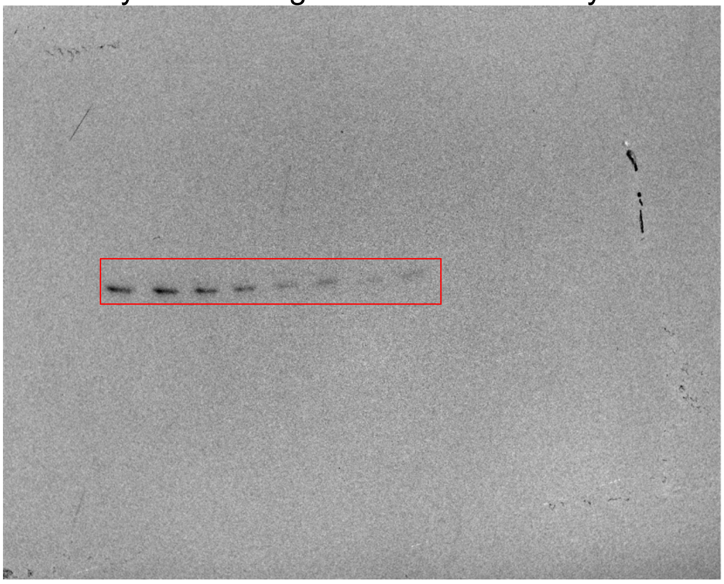

Supplementary data for Figure 1. Kinase activity of DBS1-1b

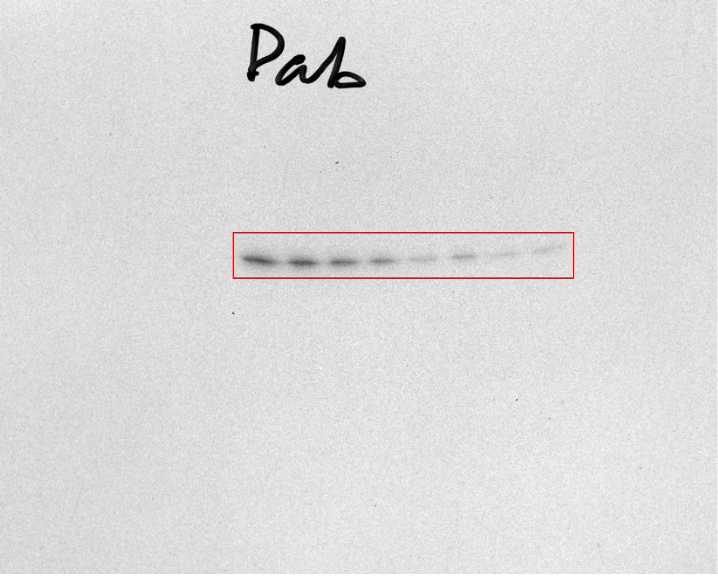

Supplementary data for Figure 1. Kinase activity of DBS1-1p

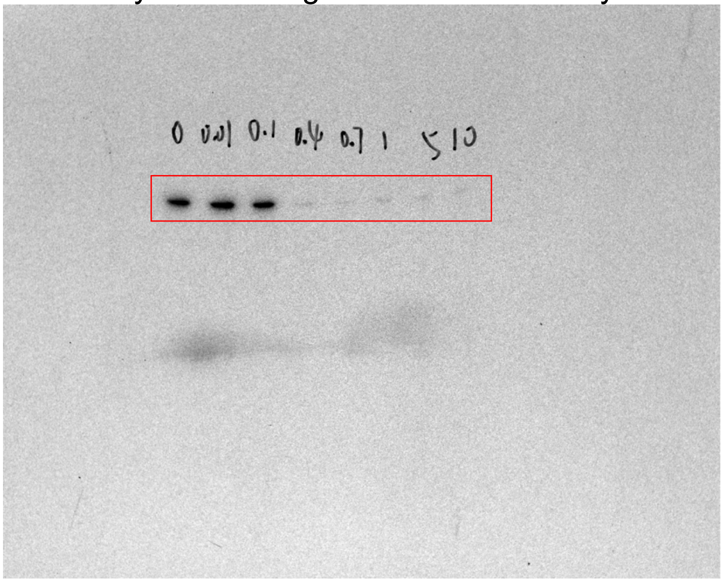

Supplementary data for Figure 1. Kinase activity of C-DBS (0.2uM)

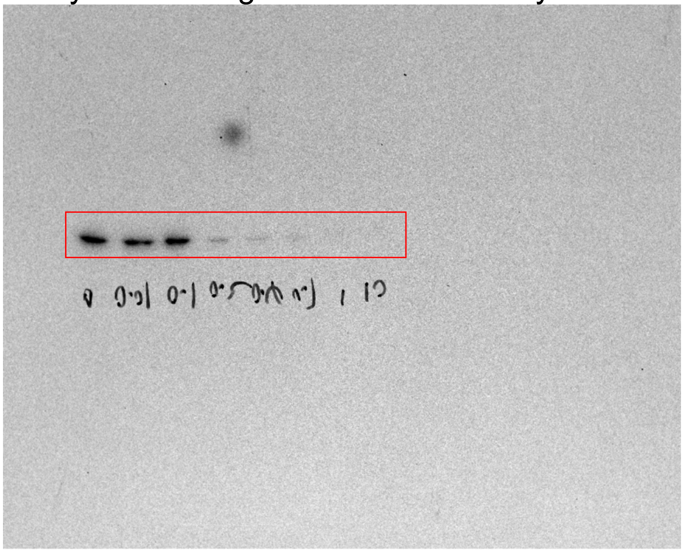

Supplementary data for Figure 1. GST pull-down, input

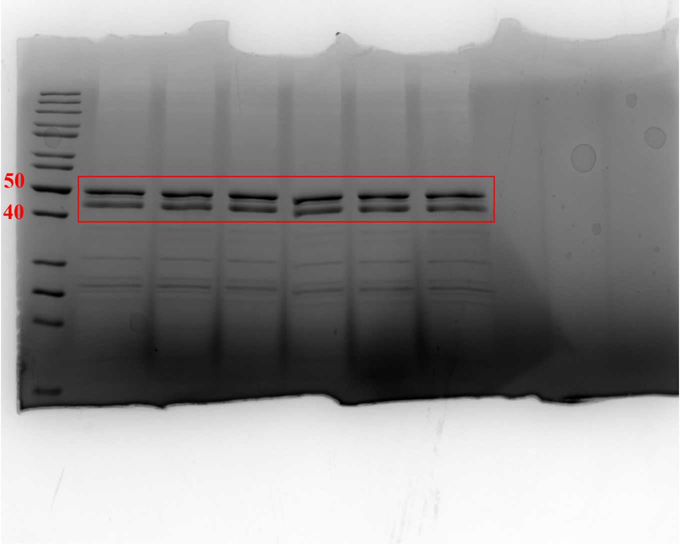

Supplementary data for Figure 1. GST pull-down, output

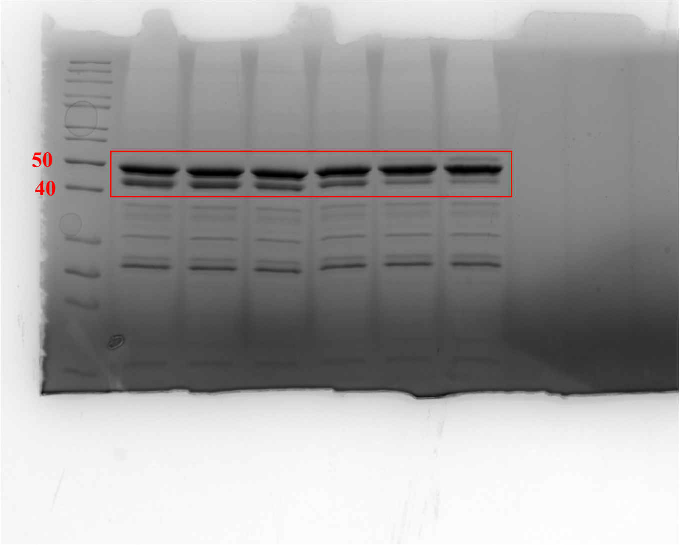

Supplementary data for Figure 2. Conjugation with SRPK1

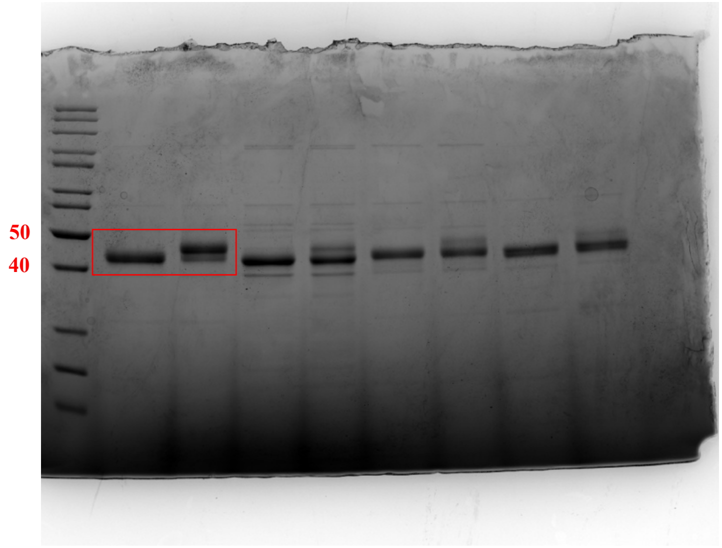

Supplementary data for Figure 2. Conjugation with SRPK1_DM

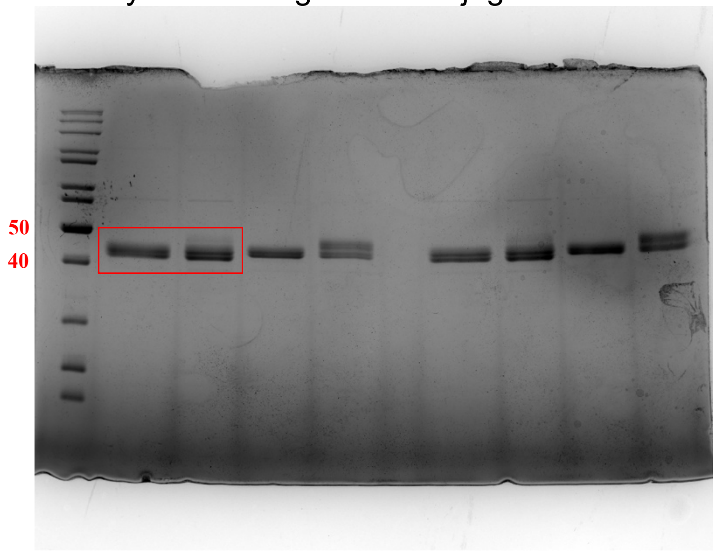

Supplementary data for Figure 2. Kinase activity assay of SRPK1_DM

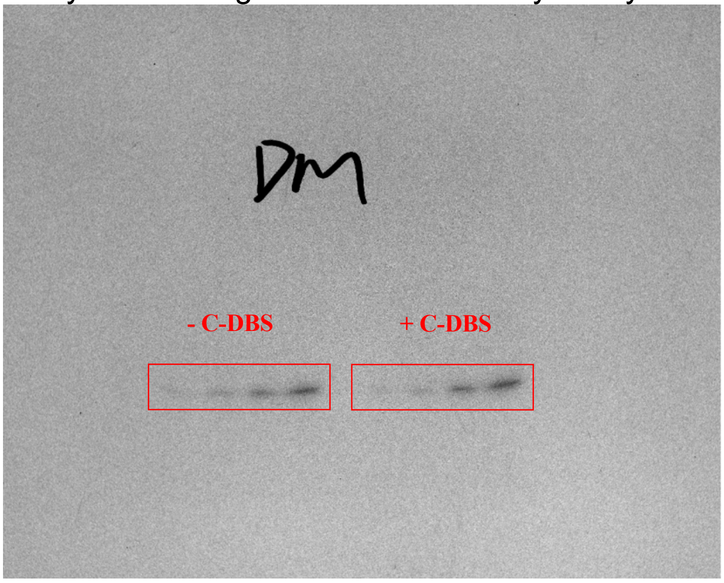

Supplementary data for Figure 3. Conjugation with SRPK1 and SRPK1_K604A

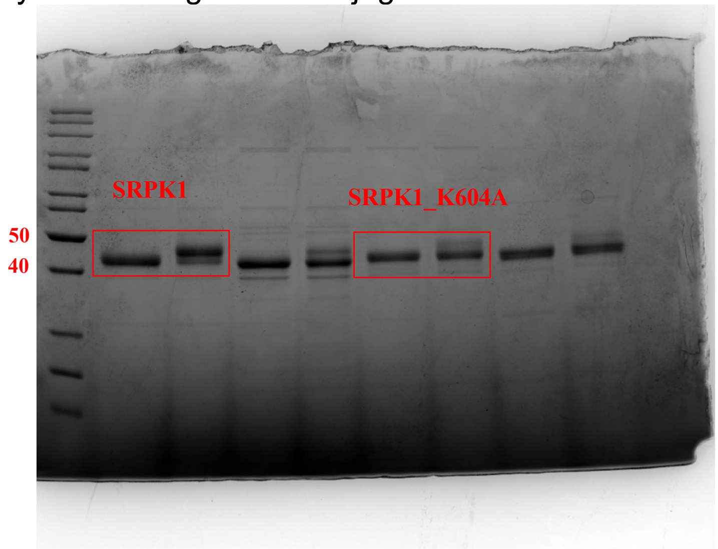

Supplementary data for Figure 4. HeLa cell lysate conjugation

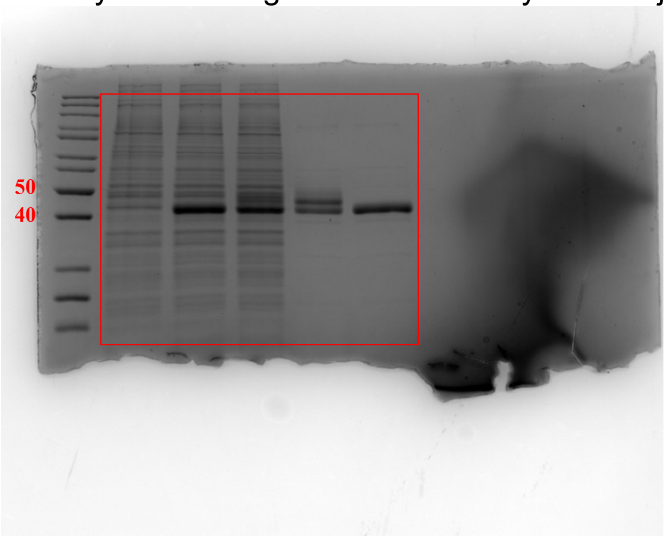

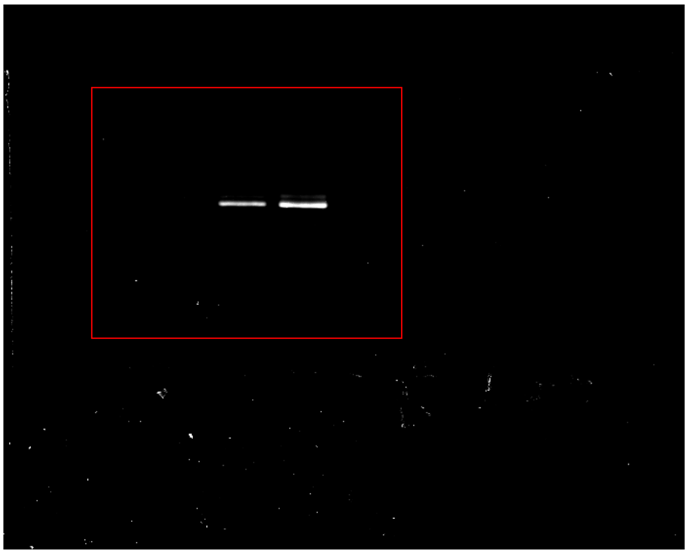

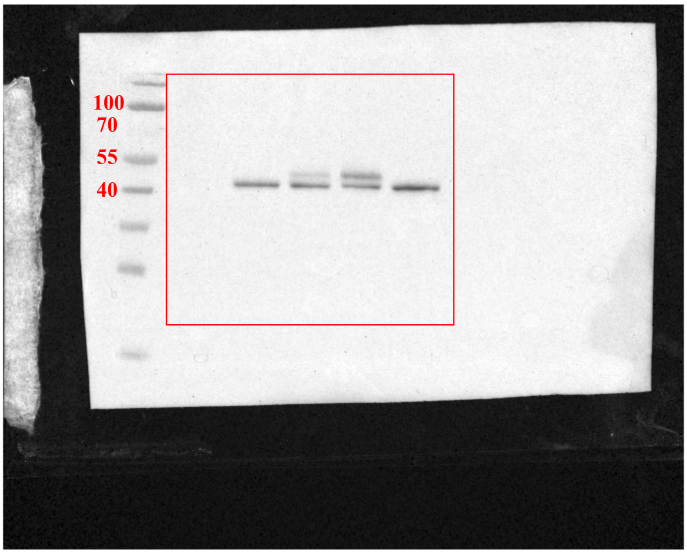

Supplementary data for Figure 4. Conjugation with MDA-MB-231 lysate

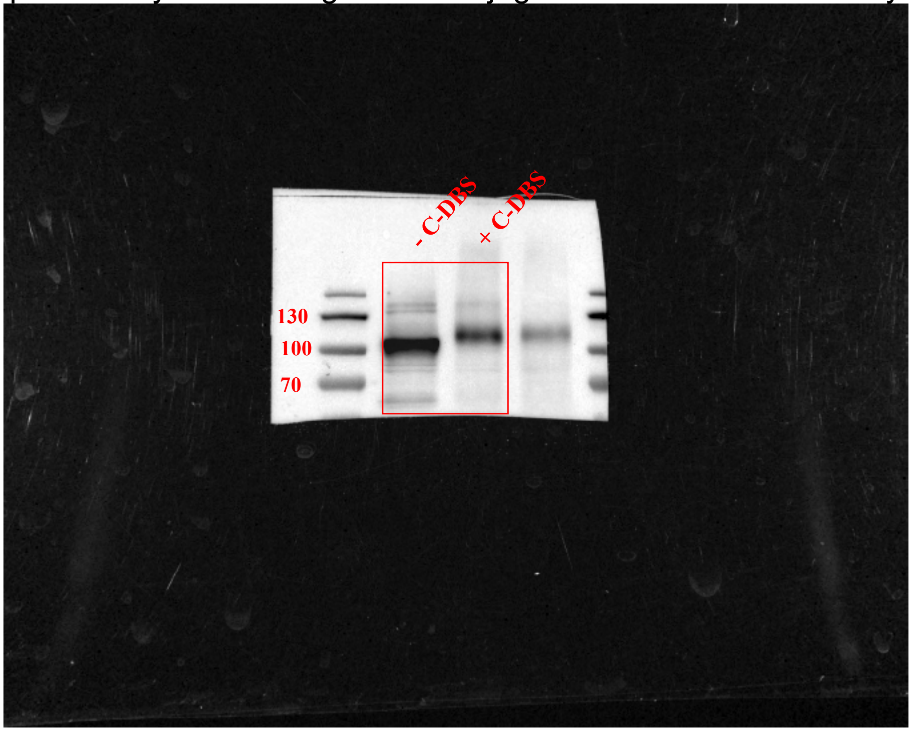

Supplementary data for Figure 4. Conjugation with SRPK2

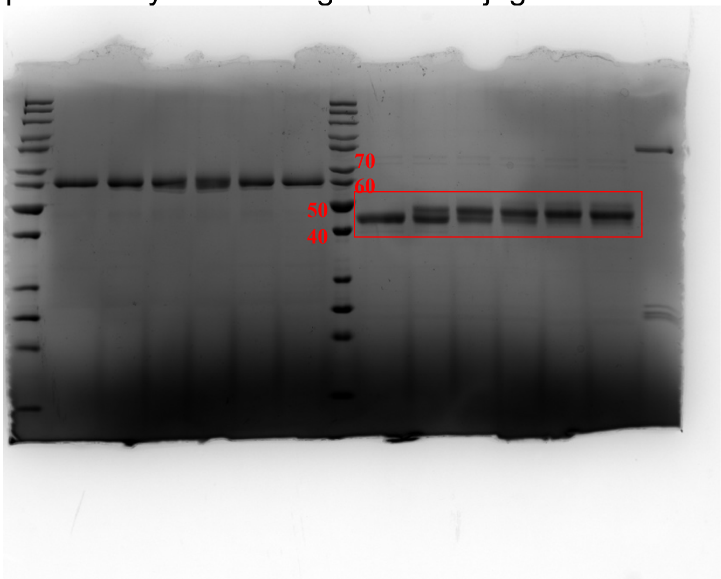

Supplementary data for Figure 4. Conjugation with CLK1(148-484)

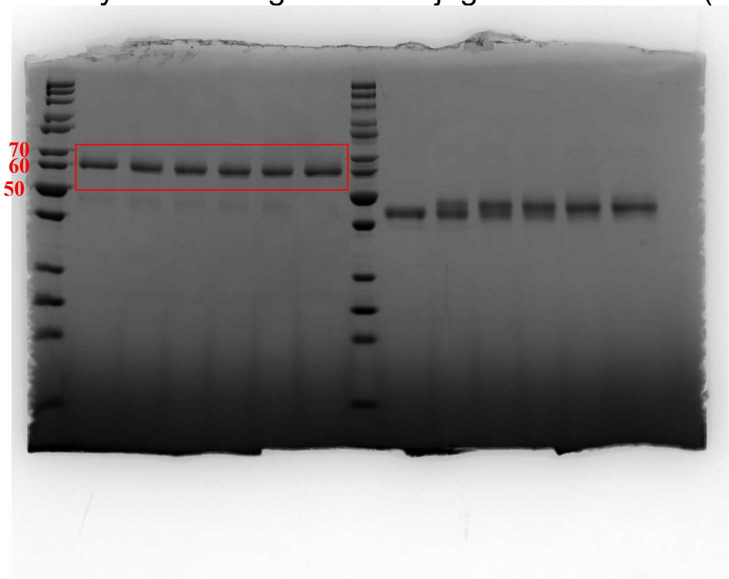

Supplementary data for Figure 4. Conjugation with BSA

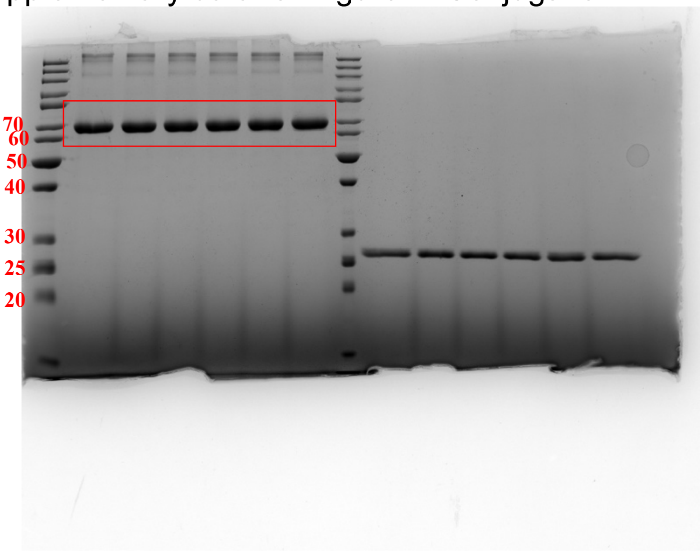

Supplementary data for Figure 4. Conjugation with GST

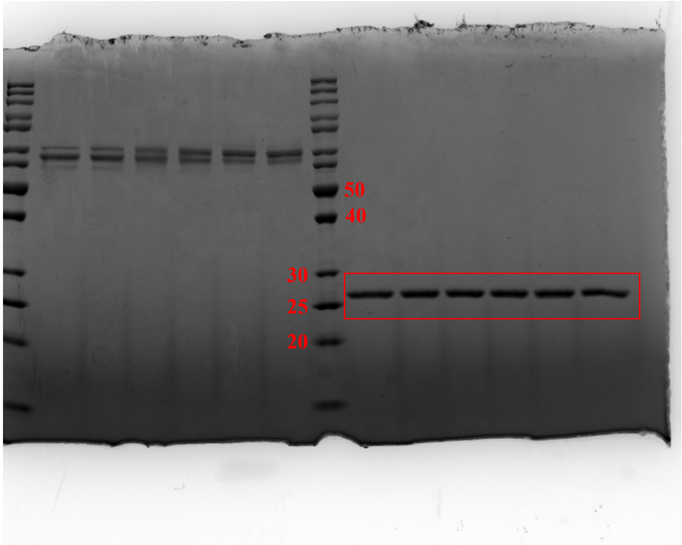

Supplementary data for Figure 4. Kinase activity of K1

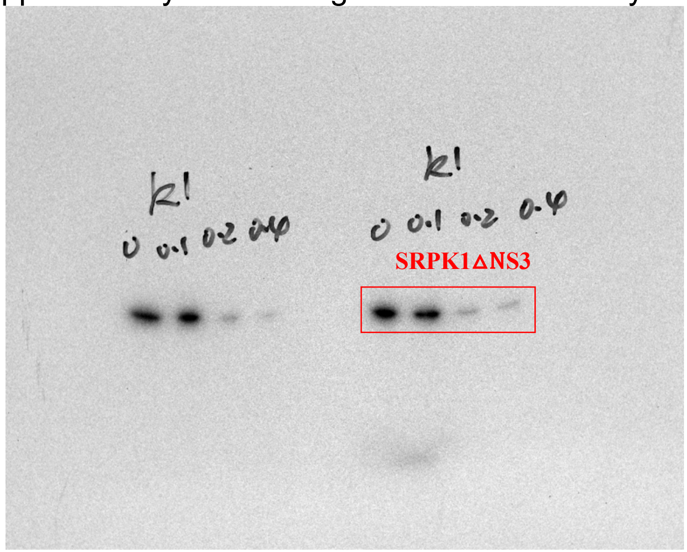

Supplementary data for Figure 4. Kinase activity of K2

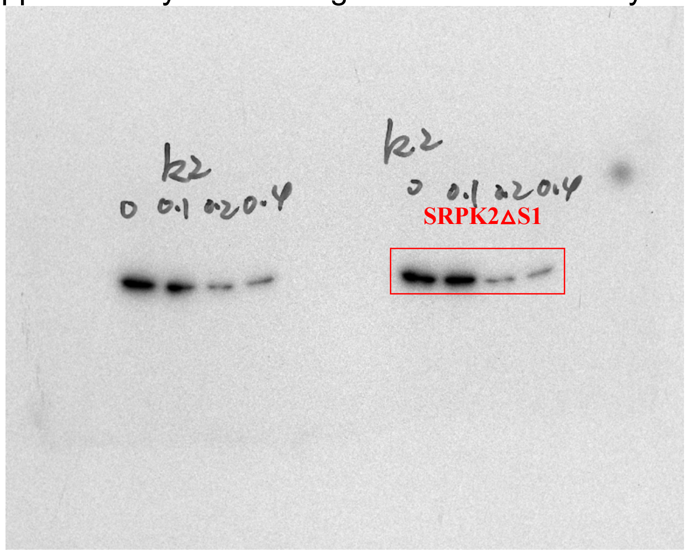

Supplementary data for Figure 4. Kinase activity of CLK and Akt

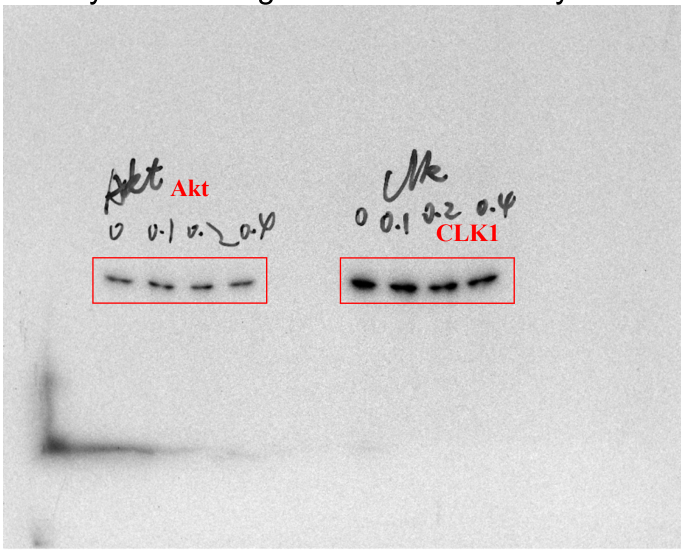

Supplementary data for Figure 6. Ab: p-SR and β-tubulin

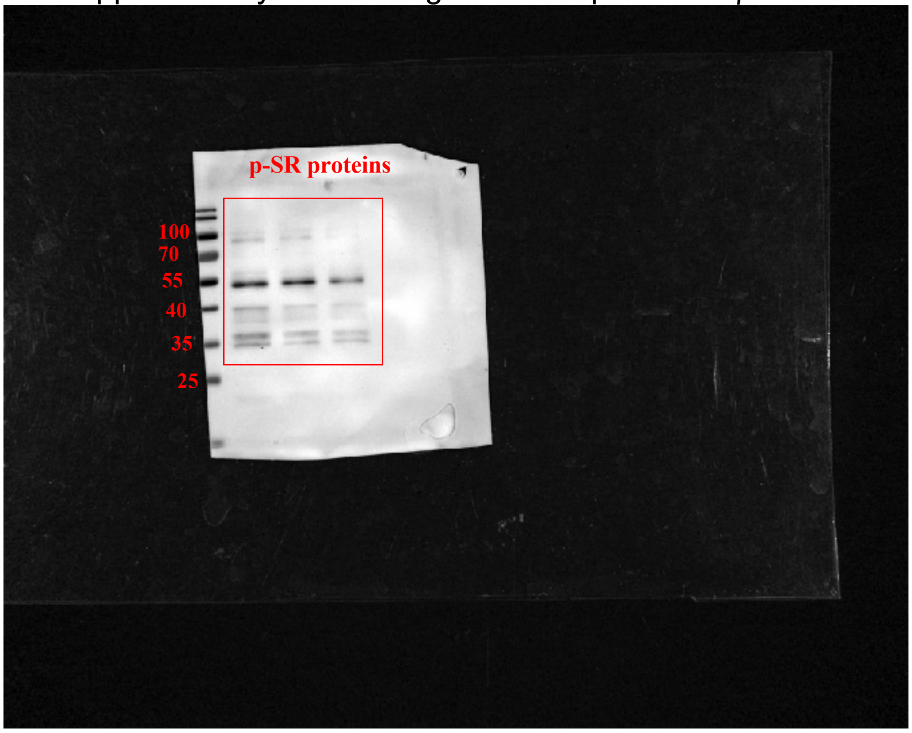

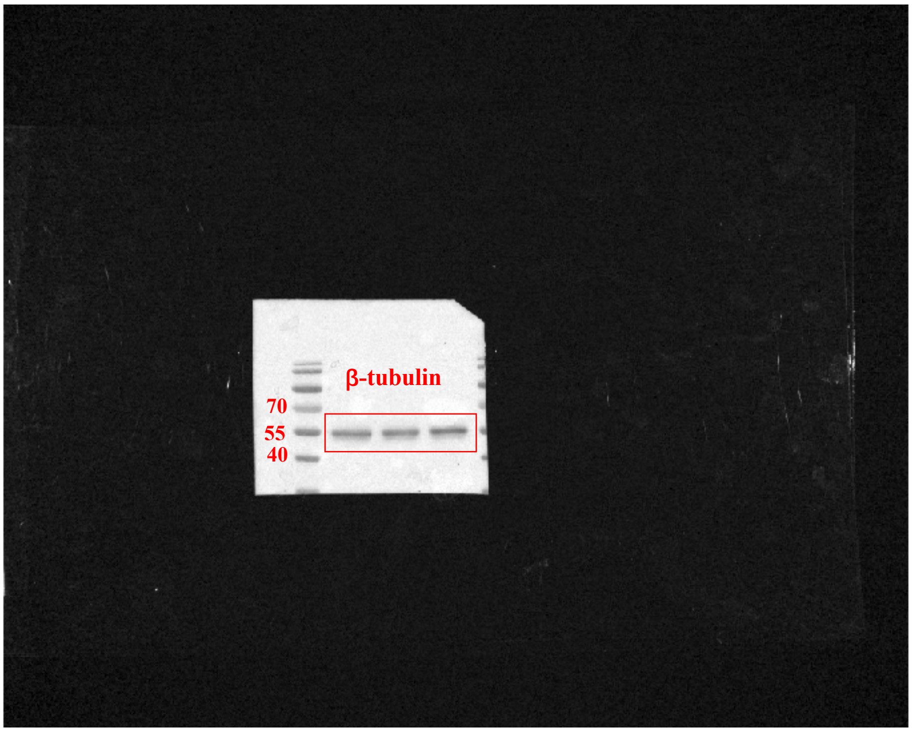

Supplementary data for Figure 7. Ab: Vimentin

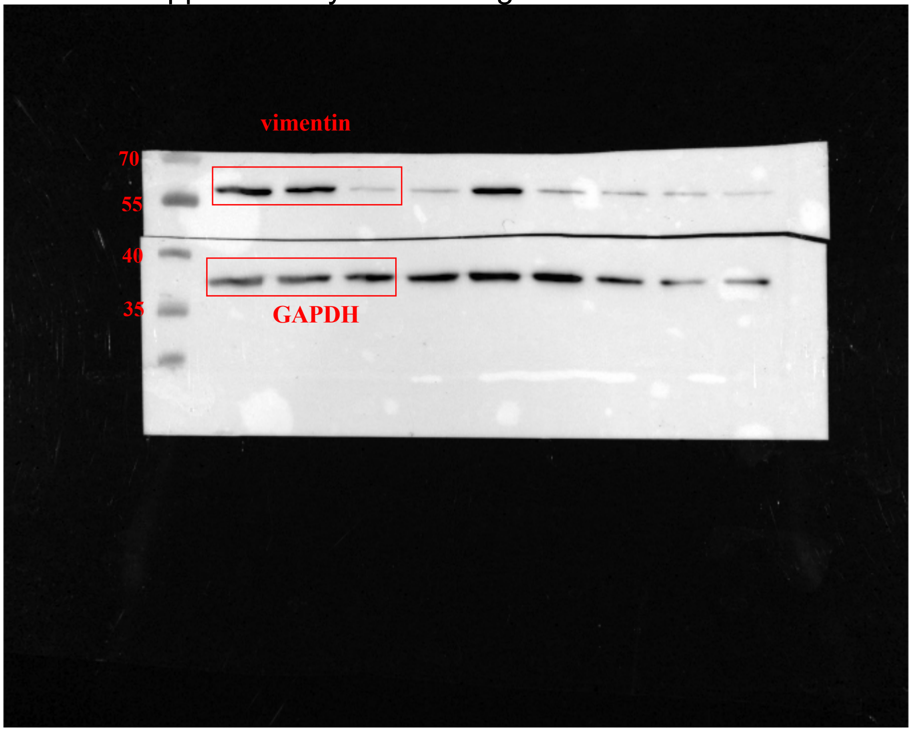

Supplementary data for Figure 7. Ab: Twist

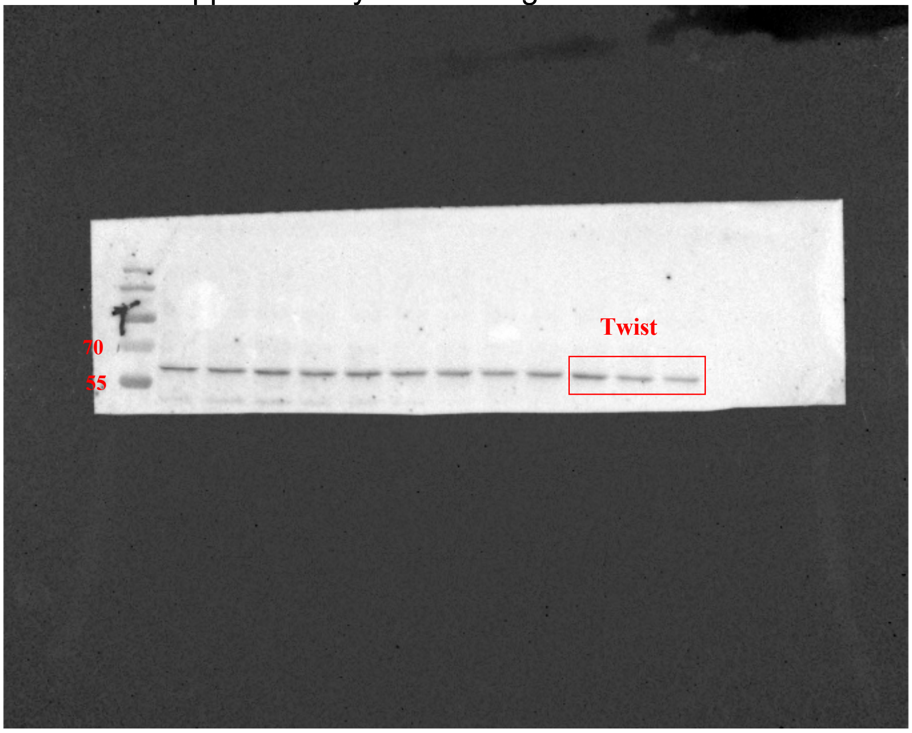

Supplementary data for Figure 7. Ab: GAPDH

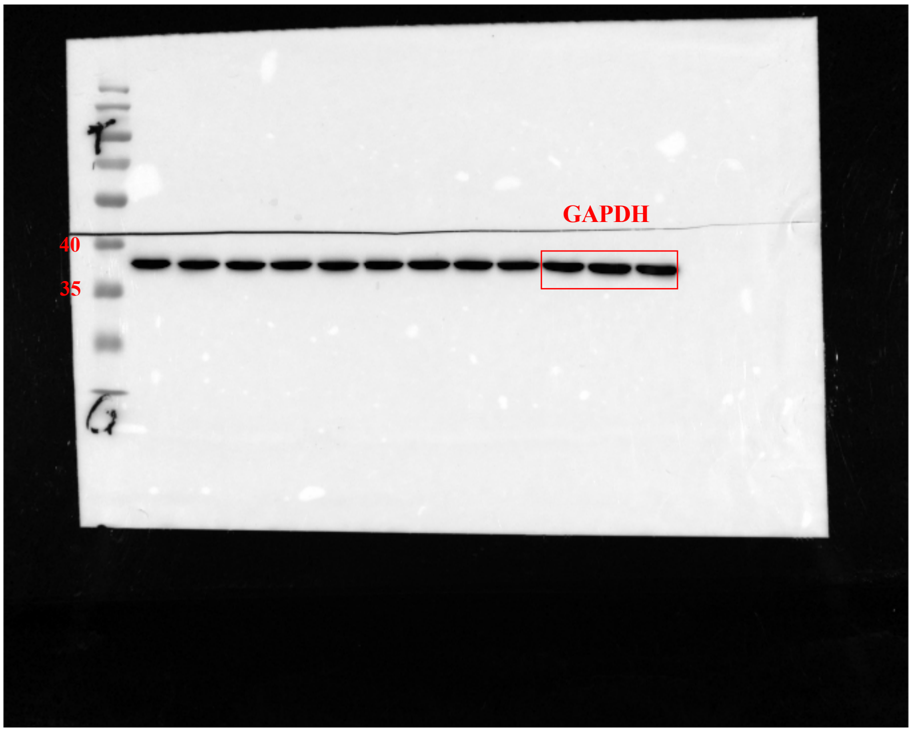

Supplementary data for Figure 7. Ab: Snail

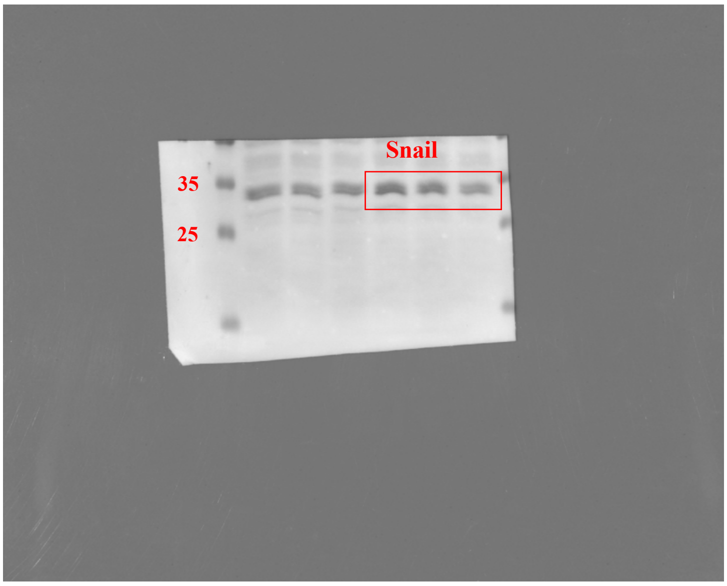

Supplementary data for Figure 7. Ab: E-cadherin, GAPDH

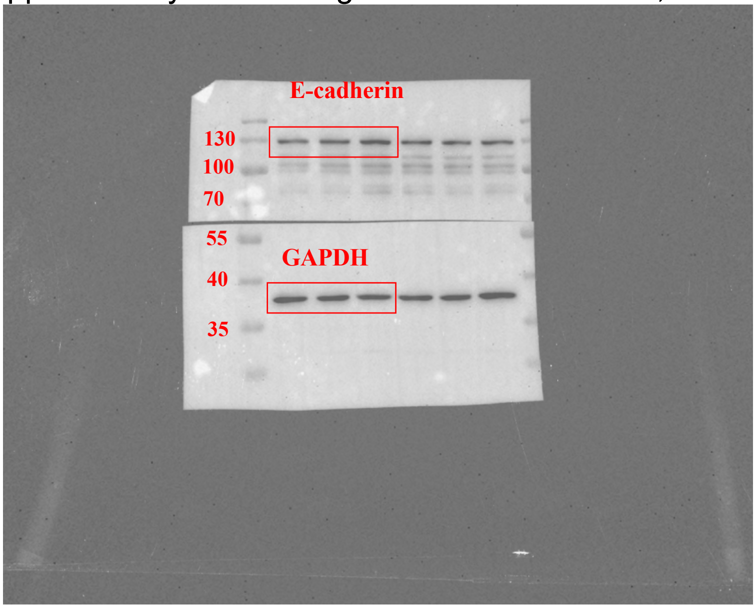

Supplement: Supplementary file 3 — Supplementary Data 1 [file 42004_2024_1230_MOESM3_ESM.pdf]
